# Mother and child health 4.5 years after gestational diabetes mellitus managed using tight or less tight targets for glycaemic control: Post-hoc follow-up study of the TARGET trial

**DOI:** 10.1371/journal.pmed.1004635

**Published:** 2026-02-03

**Authors:** Lisa J. Douglas, Greg D. Gamble, Jane E. Harding, Deborah Samuel, Carl L. Eagleton, Jane M. Alsweiler, Trecia A. Wouldes, Benjamin Thompson, Christopher J. D. McKinlay, Caroline A. Crowther

**Affiliations:** 1 Liggins Institute, University of Auckland, Auckland, New Zealand; 2 Department of Endocrinology, Greenlane Clinical Centre, Te Whatu Ora Te Toka Tumai Auckland, Auckland, New Zealand; 3 Department of Paediatrics: Child and Youth Health, University of Auckland, Auckland, New Zealand; 4 Psychological Medicine, University of Auckland, Auckland, New Zealand; 5 Department of Optometry and Vision Science, Faculty of Science, University of Waterloo, Ontario, Canada; University of Manchester, UNITED KINGDOM OF GREAT BRITAIN AND NORTHERN IRELAND

## Abstract

**Background:**

Optimal glycaemic targets for women with gestational diabetes mellitus (GDM) are unclear. The aim of this study was to compare maternal and child health 4.5 years after women with GDM had been randomised to use tight or less tight targets for glycaemic control during their pregnancy.

**Methods and findings:**

The TARGET trial was a stepped-wedge, cluster-randomised trial conducted between May 29, 2015 and November 7, 2017 at 10 hospitals in New Zealand. All hospitals were initially allocated to use less tight glycaemic treatment targets (fasting plasma glucose (FPG) <5.5 mmol/L (<99 mg/dL), 1-hour <8.0 mmol/L (<144 mg/dL), 2 hour postprandial <7.0 mmol/L (<126 mg/dL)) for women with GDM and every 4 months two hospitals were randomised to use tighter targets (FPG ≤ 5.0 mmol/L (≤90 mg/dL), 1-hour ≤7.4 mmol/L (≤133 mg/dL), 2 hour postprandial ≤6.7 mmol/L) (≤121 mg/dL). Women with GDM, blinded to the targets in use, were eligible. The primary outcome was large for gestational age. This is a post-hoc follow-up study of the TARGET randomised trial, conducted from October 2020 to June 2022. We assessed 315/427 (74%) eligible mothers and 313/427 (73%) of their children. Primary outcomes were maternal glycated haemoglobin (HbA1c) and child body mass index (BMI) z-score. Secondary outcomes included maternal cardiometabolic risk, body size, and healthcare utilisation, and for the child, body size, vision, hearing, motor function, and behavioural outcomes. Data were collected from maternal and child health questionnaires, and their health records.

Maternal HbA1c results were similar between tight and less tight glycaemic groups (40 mmol/mol standard deviation (SD) 12.6 versus 38 mmol/mol SD 8.8; adjusted mean difference (adjMD) 2.17 (95% confidence interval (CI) [−0.26, 4.60]; *P* = 0.080)). Child BMI z-scores were similar between groups (mean z-score 0.83 SD 1.72 versus 0.75 SD 1.48; adjMD 0.12 (95% CI [−0.24, 0.48]; *P* = 0.498)), although children in the tight glycaemic group were taller (107.8 cm SD 5.5 versus 106.0 cm SD 5.5; adjMD 1.83 (95% CI [0.58, 3.08]; *P* = 0.004)). Worse child outcomes were seen in the tight glycaemic group for coordination difficulties (31/109, 28.4% versus 21/118, 17.8%; adjusted relative risk (adjRR) 1.66 (95% CI [1.01, 2.73]; *P* = 0.044)), behaviour (likely on the autism spectrum 10/108, 9.3% versus 3/117, 2.6%; adjRR 3.67 (95% CI [1.02, 13.23]; *P* = 0.047)) and total difficulties scores from the strengths and difficulties questionnaire (mean score 8.4 SD 5.1 versus 6.8 SD 4.5; adjMD 1.75 (95% CI [0.51, 3.00]; *P* = 0.006)). The main limitation was the use of questionnaires rather than health professional assessments for some of the outcomes.

**Conclusions:**

Tight compared to less tight glycaemic targets in women with GDM during pregnancy did not result in lower maternal HbA1c or lower child BMI z-scores 4.5 years later, and may be associated with adverse child motor and behavioural outcomes.

## Author summary

**Why was this study done?:**

- Gestational diabetes mellitus (GDM) has significant short- and long-term health complications for the woman and her child.
- Treatment for GDM aims to reduce maternal hyperglycaemia, although it remains unclear whether tight compared to less tight glycaemic targets during pregnancy can reduce their cardiometabolic risk and improve growth and development in their children 4.5 years later.

**What did the researchers do and find?:**

- In this prospective cohort study, 315 women with GDM who had participated in a randomised trial of tight versus less tight glycaemic control, and their 313 children, were followed up 4.5 years after the birth.
- Use of tight compared with less tight glycaemic targets during pregnancy did not lower maternal HbA1c or lower child BMI z-scores.
- Children exposed to the tight targets had more adverse motor and behavioural outcomes compared to children exposed to the less tight targets.

**What do these findings mean?:**

- Further studies are needed to assess the effects on childhood health of the use of different targets for glycaemic control during pregnancy in women with GDM.
- A limitation of this study is that the adverse motor and neurodevelopmental outcomes were observed using screening questionnaires, although a health professional assessment would be needed to confirm or exclude a disorder.

## Abstract 3

In a follow-up study to a cluster randomised study, Lisa Douglas and colleagues investigated the effects of tight or less tight glycemic targets during pregnancy in women with gestational diabetes, on the health of these mothers and their children 4.5 years after birth.

## Introduction

Gestational diabetes mellitus (GDM) is a burgeoning health problem worldwide, although there is considerable variation in recommendations for many aspects of care [1]. One aspect that has received limited attention is optimal glycaemic targets for the management of GDM.

There is worldwide variation in glycaemic targets recommended for women with GDM during pregnancy [2,3]. The tight glycaemic targets in the TARGET trial [4] followed the current recommended targets in New Zealand of a fasting blood glucose concentration of ≤5.0 mmol/L (≤90 mg/dL), the lowest of all published guidelines [1]. Guidelines from the UK and USA recommend a fasting glucose <5.3 mmol/L (<95 mg/dl) [2,3]. Globally, 1-hour postprandial glucose recommendations vary between ≤7.4 and 7.8 mmol/L (133–141 mg/dl), and 2-hour postprandial glucose recommendations between <6.4 and 6.7 mmol/L (115–121 mg/dl) [2,3].

The relevant Cochrane review [5] on glycaemic targets for the treatment of GDM included 1,731 women from four randomised trials, one of which was the TARGET trial [4]. The evidence available for review was considered to be of low quality [5]. There were no differences between tight and less tight glycaemic groups for caesarean section and induction of labour, nor for the infant outcomes of large for gestational age, hypoglycaemia, and perinatal mortality. In the tight glycaemic group, hypertensive disorders of pregnancy may have been more likely (relative risk (RR) 1.16 (95% confidence interval (CI) [0.80, 1.69])), and the composite of perinatal mortality or serious morbidity was reduced (RR 0.84 (95% CI [0.55, 1.29])) [5].

GDM is associated with later adverse cardiometabolic outcomes for mother and child [6]. The Hyperglycemia and Adverse Pregnancy Outcomes (HAPO) follow-up study reported that mothers with a history of GDM were more likely to develop a glucose metabolism disorder than those without, at a median follow-up time of 11.4 years (52.2% versus 20.1%) [6]. In their children, no association between GDM and being overweight or obese was found after adjusting for maternal body mass index (BMI), although there was an association with childhood obesity alone, and other measures of childhood adiposity including increased body fat, skinfolds, and waist circumference.

The TARGET trial was a stepped-wedge, cluster-randomised trial comparing tight with less tight glycaemic targets during pregnancy [4]. Tight compared with less tight glycaemic targets did not reduce the risk of a large for gestational age infant but did reduce serious infant morbidity, although serious maternal morbidity was increased. The primary aim of this TARGET 4.5 Year Follow-Up Study was to assess whether recommendation for use of tight targets for glycaemic control during pregnancy for mothers with GDM compared with less tight targets reduced their later cardiometabolic risk and improved growth and development of their children. Our hypothesis was that use of tight glycaemic targets would result in improved maternal and childhood outcomes when compared with less tight glycaemic targets.

## Methods

### Study design and participants

In the TARGET trial, registered in the Australian New Zealand Clinical Trials Registry (ACTRN 12615000282583), 10 participating hospitals in New Zealand were randomised to when they changed from using less tight glycaemic targets for treating women with GDM (fasting blood glucose <5.5 mmol/L (<99 mg/dL), 1 hour postprandial <8 mmol/L (<144 mg/dL), and 2 hour postprandial <7 mmol/L (<126 mg/dL)) to using tight glycaemic targets (fasting blood glucose ≤5 mmol/L (≤90 mg/dL), 1 hour postprandial ≤7.4 mmol/L (≤133 mg/dL), and 2 hour postprandial ≤6.7 mmol/L (≤121 mg/dL)) [4]. A waiver of consent applied for eligible women attending the participating hospitals to be included in the trial if an oral glucose tolerance test (OGTT) had confirmed a diagnosis of GDM between 22 and 34 weeks’ gestation and their foetus did not have a known major malformation. Women with GDM were cared for by their lead maternity carer and the local Diabetes in Pregnancy service according to standard practice at each hospital that included diet and lifestyle advice, blood glucose monitoring, and pharmacological treatment as needed [4].

The TARGET 4.5 Year Follow-Up Study was approved by the Northern B Health and Disability Ethics Committee (20/NTB/33). All mothers with a singleton pregnancy who had given written consent for additional in-depth assessments in the TARGET trial, and their children, were eligible for this follow-up study if they had not withdrawn from further follow-up, and were known to be alive. If the child was in care outside of the family both mother and child were not eligible for follow-up. This study is reported as per the Consolidated Standards of Reporting Trials (CONSORT) guidelines (S1 Checklist).

### Randomisation and masking

Hospital clusters were randomised to the date at which they changed from using the less tight to the tight glycaemic targets with the sequence determined by a computer-generated random number table [4]. Researchers were blinded to the pregnancy and postnatal history of mother and child.

### Procedures

Contact tracing was performed using the TARGET trial database after checks were made to ensure families were not contacted if their child had died. An invitation letter and participant information sheet were sent to the mother two months before the child turned 4.5 years of age, inviting them to contact the study team. If no response was received after 1–2 weeks a phone call was made to ensure they had received the information and to discuss the study. Those who gave consent were asked to complete the study’s maternal health and well-being and child health and developmental questionnaires. Blood test results closest to 4.5 years after birth were retrieved from the mother’s medical records (glycated haemoglobin (HbA1c), plasma lipids, and fasting blood glucose), as were their child’s Before School Check (B4SC) pre-school health screening results [7].

### Outcomes

The primary outcome for the mothers was HbA1c and for the children BMI z-score [8] 4.5 years after the birth. Maternal secondary outcomes were a diagnosis of Type 2 diabetes (HbA1c ≥ 50 mmol/mol (≥6.7%) on at least two occasions or self-reported diabetes), pre-diabetes (HbA1c 41−49 mmol/mol (5.9%−6.6%), or self-reported pre-diabetes) [9], body size (height, weight, and BMI), plasma lipids, hypertension, and a diagnosis of metabolic syndrome [10,11]. A blinded review of HbA1c results by an adjudication panel identified the most appropriate result to include in the analysis, and the diagnosis of type 2 diabetes, pre-diabetes, and metabolic syndrome. HbA1c data were considered missing if collected <3 years or >6 years after the TARGET birth. Self-reported questionnaires recorded any further pregnancies (number and if complicated by GDM or high blood pressure or pre-eclampsia or preterm birth), healthcare utilisation (prescriptions for the treatment of diabetes, hypertension and dyslipidemia, cardiovascular or cerebrovascular disease, and other major health conditions), health related quality of life (SF-36 version 2) [12], vulnerability to depression (Edinburgh Postnatal Depression Score (EPDS) with scores ≥12 indicating likely suffering from depression) [13], and anxiety (short-form Spielberger State-Trait Anxiety Inventory (STAI) with scores ≥15 indicating abnormal levels of anxiety) [14]. Data on the prespecified outcomes of adherence to postpartum diabetes screening recommendations, maternal dietary macronutrients, and maternal physical activity patterns will be reported in separate manuscripts.

Childhood secondary outcomes were overweight or obese (BMI z-score >2) [8], overweight (BMI z-score >2 and <3), obese (BMI z-score ≥3) [15], height and weight and their z-scores, short stature (height z-score < −2), underweight (weight z-score < −2), and normal weight (weight z-score −2–2) [15], neurological status (a series of questions to assess for cerebral palsy, developmental delay, coordination delay, and visual problems and hearing problems), fine and gross motor function (The Little Developmental Coordination Disorder Questionnaire (Little DCDQ), scores <67 for boys and <68 for girls indicating coordination difficulty) [16], behavioural and emotional problems (Strengths and Difficulties Questionnaire (SDQ), scores ≥14 indicating behavioural difficulty) [17,18], communication (Social Communication Questionnaire (SCQ) scores ≥15 indicating likely on the autism spectrum) [19,20], eating behaviour (Child Eating Behaviour Questionnaire (CEBQ)) [21], and functional health and wellbeing (Child Health Questionnaire (CHQ PF-50) with scores more than 1 standard deviation (SD) below the mean considered abnormal) [22]. Ethnicity was self-reported and prioritised according to the New Zealand Ministry of Social Development [23]. Socioeconomic status was assigned using the New Zealand Deprivation Index, with 10 the highest and 1 the lowest neighbourhood deprivation level [24].

### Statistical analysis

All analyses were performed using SAS software version 9.4 or later (SAS Institute, Cary, NC, USA) following the statistical analysis plan. Prior to unblinding the proportion of participants in each step-wedge cluster was examined. As there were considered to be too few participants for analysis according to the step-wedge design of the TARGET trial, the primary analysis was an independent groups analysis without adjustment for clustering and time of randomisation on the basis that the models were unlikely to converge with sparse intra-cluster data. For primary outcomes only, a post-hoc cluster analysis was performed to account for clustering within pairs of hospitals within each wedge and for the time of stepping in the primary stepped-wedge trial.

Assuming an equal variance and a mean HbA1c in the less tight target group of 35 mmol/mol (5.4%) (SD 4.4) based on the booking HbA1c of mothers in the TARGET trial, with 315 mothers a difference of at least 1.6 mmol/mol (0.2%) (SD 0.36) between the less tight and tight target groups could be detected with 90% power at the 5% significance level using PASS 16 [25]. With 313 children differences of 0.7 kg/m^2^ (SD 0.36) could be detected, based on BMI at 4.5 years (mean 16.5 kg/m^2^, SD 2.0) of 140 children of mothers with GDM born at Waikato Hospital who participated in a study of neonatal hypoglycaemia [26].

The analyses were performed blinded to the glycaemic target allocation. An intention-to-treat approach was used in which participants were analysed according to the treatment target their hospital was randomised to when they received care. Both unadjusted and adjusted analyses were performed for the pre-defined covariate of gestational age (GA) at time of the OGTT. The primary outcomes were judged to be independent and therefore we did not adjust for multiplicity. Statistical significance was assessed using a two-sided comparative test of treatment effect, comparing the tight to the less tight glycaemic target group, using an α value of 0.05.

For children for whom the only weight or height measurement recorded was when they were younger than 3 years or older than 6 years, these data were considered missing. We assessed the overall pattern of missing data, amount and distribution between groups and outcomes. No imputation for child height or weight was performed.

Continuous outcomes were analysed using a generalised linear mixed-effects model (GLMM) fitting a normal distribution and identity link function. Marginal ‘least squares’ mean difference [27] and 95% CIs were estimated from the adjusted model (adjusted mean difference). All model assumptions, including normality, were assessed. Binary outcomes were analysed using a GLMM fitting a binary distribution and log link function to estimate unadjusted relative risk and adjusted relative risk with robust 95% CIs.

A post-hoc nonparametric sensitivity analysis of the continuous child neurodevelopmental outcomes was performed using median quantile regression at the 50th percentile (QUANTREG procedure of SAS) with adjustment for gestational age at the time of oral glucose tolerance test. Confidence intervals were calculated using the Koenker and Bassett rank-based (inversion method) [28].

Exploratory analyses were performed for the primary and secondary maternal and child outcomes, adjusting for the additional covariates of gestation at birth (preterm/term), maternal ethnicity, pharmacological treatment of GDM, trial cluster (hospital pairs), socioeconomic status, and for the child outcomes only neonatal hypoglycaemia.

## Results

**Fig 1:**
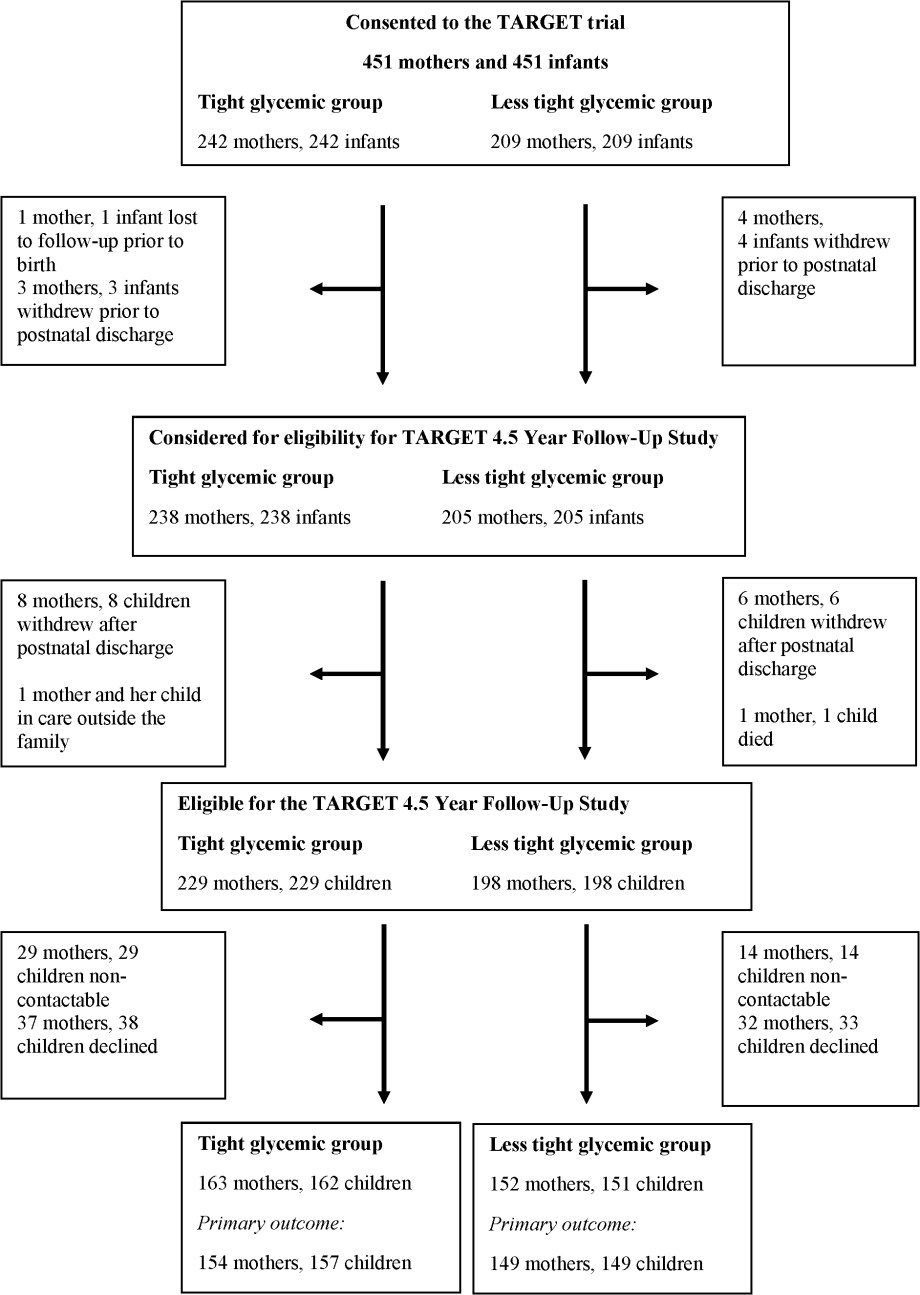
Flowchart of participants in the TARGET 4.5 Year Follow-Up Study.

Of the 451 mothers with a singleton pregnancy who consented to additional assessments in the TARGET trial, 427 mothers and their children were eligible for the 4.5 year follow-up study; 229 mothers and children in the tight glycaemic target group and 198 mothers and children in the less tight glycaemic target group (Fig 1). In the tight target group there were 163 mothers (71% of those eligible) and 162 children (71% of those eligible) who participated in the follow-up study and in the less tight target group 152 mothers (77% of those eligible) and 151 children (76% of those eligible) (Fig 1). The follow-up study was conducted between 6th October 2020 and 10th May 2022.

Maternal characteristics were similar between eligible mothers who did and did not participate in this study, although participants were less likely to be severely deprived (89/300, 29.7% versus 51/109, 46.8%) and more likely to be New Zealand European (152/315, 48.3% versus 31/112, 27.7%). Children who participated were more likely to be female than children who did not participate (169/313, 54.0% versus 45/113, 39.8%) (S1 Table).

Mothers who were eligible for inclusion in the TARGET 4.5 Year Follow-Up study were similar at entry to the TARGET trial in the tight and less tight glycaemic groups, although a higher proportion of Pacific mothers and a lower proportion of New Zealand European mothers were allocated to the tight group (29/163, 17.8% versus 11/152, 7.2% and 66/163, 40.5% versus 86/152, 56.6%, respectively). Mothers in the tight glycaemic group were more likely to receive pharmacological treatment for GDM (120/163, 73.6% versus 97/152, 63.8%) compared with mothers in the less tight glycaemic group. Child age at follow-up was similar in both glycaemic groups at a mean 4.4 years, SD 0.40 (Table 1).

**Table 1:** Characteristics of mothers and children included in the 4.5 Year Follow-Up.

Mothers	Tight glycaemic group	No. *n* = 163	Less tight glycaemic group	No. *n* = 152
Maternal age at birth, years	32.9 (5.1)	163	33.5 (4.8)	152
Primiparity, *n*(%)	64 (39.3)	163	61 (40.1)	152
Gestational age at entry to TARGET trial (weeks)^*^	27.5 (26.2, 29.0)	163	27.3 (25.8, 28.4)	152
BMI at entry to TARGET trial (kg/m^2^)	32.5 (7.4)	161	32.3 (7.1)	151
Underweight (<18.5 kg/m^2^), *n*(%)	0 (0)	161	0 (0)	151
Normal weight (18.5–24.9 kg/m^2^), *n*(%)	13 (8.1)	161	16 (10.6)	151
Overweight (25.0–29.9 kg/m^2^), *n*(%)	51 (31.7)	161	52 (34.4)	151
Obese (≥30 kg/m^2^), *n*(%)	97 (60.2)	161	83 (55.0)	151
Previous history of GDM prior to index pregnancy, *n*(%)	27 (16.5)	163	33 (21.7)	152
Smoking at trial entry, *n*(%)	10 (6.1)	163	12 (7.9)	152
Prioritised ethnicity^†^				
Māori, *n* (%)	15 (9.3)	163	10 (6.6)	152
Pacific, *n*(%)	29 (17.8)	163	11 (7.2)	152
NZ European, *n*(%)	66 (40.5)	163	86 (56.6)	152
Asian, *n*(%)	51 (31.2)	163	41 (27.0)	152
Other, *n*(%)	2 (1.2)	163	4 (2.6)	152
NZ Deprivation Category^‡^				
1–2, *n*(%) – least deprived	19 (12.3)	154	26 (17.8)	146
3–4, *n*(%)	24 (15.6)	154	32 (21.9)	146
5–6, *n*(%)	22 (14.3)	154	22 (15.1)	146
7–8, *n*(%)	35 (22.7)	154	31 (21.2)	146
9–10, *n*(%) – most deprived	54 (35.1)	154	35 (24.0)	146
OGTT plasma glucose concentrations at trial entry				
Fasting glucose (mmol/l)^*^	5.2 (4.6, 5.7)	163	5.1 (4.4, 5.6)	152
2 hour glucose (mmol/l)^*^	9.5 (9.0, 10.2)	163	9.6 (9.1, 10.2)	152
Pharmacological treatment for GDM in index pregnancy	120 (73.6)	163	97 (63.8)	152
**Children**	**Tight glycaemic group**	***n* = 162**	**Less tight glycaemic group**	***n* = 151**
Gestational age at birth, weeks^*^	38.6 (37.7, 39.1)	162	38.6 (38.0, 39.3)	151
Male, *n*(%)	70 (43.2)	162	74 (49.0)	151
Birthweight (g)	3,283 (494)	162	3,340 (561)	151
LGA, *n*(%)	17 (10.5)	162	22 (14.6)	151
SGA, *n*(%)	8 (4.9)	162	10 (6.6)	151
Neonatal hypoglycaemia, *n*(%)	50 (30.9)	162	36 (23.8)	151
Prioritised ethnicity^†^				
Māori, *n*(%)	16 (9.9)	162	9 (6.0)	151
Pacific, *n*(%)	29 (17.9)	162	11 (7.3)	151
NZ European. *n*(%)	52 (32.1)	162	75 (49.7)	151
Asian, *n*(%)	49 (30.2)	162	41 (27.2)	151
Other, *n*(%)	16 (9.9)	162	15 (9.9)	151
Age at time of follow-up, years	4.4 (0.4)	162	4.4 (0.4)	151

Data are mean (standard deviation, SD) unless otherwise indicated. No. = number of participants providing information for that outcome.

*median (interquartile range). OGTT = oral glucose tolerance test. LGA = large for gestational age, defined as >90th centile. SGA = small for gestational age, defined as <10th centile.

†Ethnicity was determined by self-report, according to the New Zealand Ministry of Social Development and prioritised for analysis [23].

‡NZ Deprivation Category determined using the NZ Deprivation Index (NZDEP) [24].

The primary outcome for the mother of HbA1c was similar in the tight glycaemic group compared with the less tight glycaemic group (HbA1c 40 mmol/mol SD 12.6 versus 38 mmol/mol SD 8.8; adjusted mean difference (adjMD) 2.17 mmol/mol (95% CI [−0.26, 4.60]; *P* = 0.080)) (Table 2). The maternal HbA1c results remained similar in exploratory analyses after the prespecified additional adjustment for gestational age at birth (adjMD 2.03 mmol/mol (95% CI [−0.40, 4.45]; *P* = 0.102)), maternal ethnicity (adjMD 0.62 mmol/mol (95% CI [−1.73, 2.96]; *P* = 0.607)), use of pharmacological treatment for GDM (adjMD 1.41 mmol/mol (95% CI [−1.00,3.83]; *P* = 0.249)), trial cluster (adjMD 0.66 mmol/mol (95% CI [−2.00,3.33]; *P* = 0.623)) and socioeconomic status (adjMD 1.60 mmol/mol (95% CI [−0.96,4.15]; *P* = 0.220)) (S2 Table).

**Table 2:** Primary and secondary outcomes among the mothers at 4.5 Year Follow-Up.

	Tight glycaemic group	No. *n* = 163	Less tight glycaemic group	No. *n* = 152	Unadjusted treatment effect [95% CI]	Treatment effect adjusted for GA OGTT only [95% CI]	*P* value
Primary outcome							
Maternal HbA1c (mmol/mol)	40 (12.6)	154	38 (8.8)	149	1.67 [−0.80, 4.14]	2.17 [−0.26, 4.60]	0.080
Maternal HbA1c (%)	5.8 (1.6)	154	5.6 (0.8)	149	0.15 [−0.07, 0.38]	0.20 [−0.02, 0.42]	0.080
Secondary Outcomes							
Any of pre-diabetes or diabetes, *n*(%)^*^	47 (28.8)	163	46 (30.3)	152	0.95 [0.68, 1.34]	1.02 [0.73, 1.41]	0.929
Prescriptions to treat pre-diabetes or diabetes, *n*(%)^†^	13 (27.7)	47	17 (37.0)	46	0.75 [0.41, 1.37]	0.78 [0.43, 1.42]	0.417
Pre-diabetes, *n*(%)^*^	37 (22.7)	163	36 (23.7)	152	0.95 [0.64, 1.42]	1.01 [0.68, 1.49]	0.938
Prescriptions to treat pre-diabetes, *n*(%)^†^	4 (10.8)	37	7 (19.4)	36	0.56 [0.17, 1.77]	0.61 [0.19, 1.96]	0.404
Type 2 diabetes, *n*(%)^*^	10 (6.1)	163	10 (6.6)	152	0.93 [0.40, 2.19]	1.07 [0.46, 2.50]	0.875
Prescriptions to treat type 2 diabetes, *n*(%)^†^	9 (90.0)	10	10 (100.0)	10	NE	NE	NE
Height (cm)	162.6 (6.4)	163	162.5 (7.0)	152	0.15 [−1.33, 1.63]	0.01 [−1.47, 1.50]	0.984
Weight (kg)	82.0 (24.6)	163	82.9 (24.1)	152	−0.89 [−6.29, 4.52]	−0.55 [−5.98, 4.88]	0.843
BMI (kg/m^2^)	30.9 (8.5)	163	31.2 (8.0)	152	−0.34 [−2.18, 1.49]	−0.16 [−2.00, 1.68]	0.864
Plasma lipids							
Total cholesterol (mmol/L)	5.1 (1.0)	86	5.0 (0.9)	85	0.08 [−0.22, 0.37]	0.02 [−0.28, 0.32]	0.885
Triglycerides (mmol/L)^*^	1.8 (0.8)	86	1.6 (0.7)	85	0.24 [0.01, 0.48]	0.25 [0.01, 0.49]	0.039
HDL result (mmol/L)^*^	1.3 (0.3)	86	1.4 (0.4)	85	−0.07 [−0.17, 0.03]	−0.08 [−0.19, 0.02]	0.105
LDL result (mmol/L)	3.1 (0.8)	85	3.0 (0.9)	84	0.04 [−0.22,0.30]	−0.01 [−0.27,0.25]	0.912
Total cholesterol/HDL ratio	4.1 (1.1)	86	3.8 (1.1)	85	0.27 [−0.06,0.60]	0.26 [−0.07,0.59]	0.127
Dyslipidemia, *n*(%)	19 (13.9)	137	21 (16.7)	126	0.83 [0.47, 1.48]	0.84 [0.47, 1.49]	0.547
Hypertension, *n*(%)	17 (12.3)	138	19 (14.8)	128	0.83 [0.45, 1.53]	0.84 [0.45, 1.55]	0.567
Previous cardiovascular event, *n*(%)	0	138	0	128	NE	NE	NE
Previous cerebrovascular event, *n*(%)	1 (0.7)	138	1 (0.8)	128	0.93 [0.06, 14.86]	0.92 [0.06, 15.22]	0.956
Metabolic syndrome, *n*(%)^*^	32 (19.6)	163	31 (20.4)	152	0.96 [0.62, 1.50]	1.01 [0.65, 1.57]	0.950
Any further pregnancies, *n*(%)	50 (36.0)	139	52 (39.7)	131	0.91 [0.67, 1.23]	0.90 [0.66, 1.23]	0.512
Mean number of further pregnancies	1.3 (0.6)	139	1.4 (0.8)	131	−0.85 [−0.5, 0.18]	−0.11 [−0.38, 0.15]	0.390
Number of further pregnancies with GDM, *n*(%)	28 (56.0)	50	25 (48.1)	52	1.14 [0.80, 1.64]	1.23 [0.88, 1.72]	0.225
Number of further pregnancies with pre-eclampsia, *n*(%)	4 (8.0)	50	5 (9.6)	52	0.85 [0.24, 3.03]	0.84 [0.23, 3.04]	0.785
Number of further pregnancies with pre-term birth, *n*(%)	7 (14.0)	50	8 (15.4)	52	0.96 [0.38, 2.45]	0.99 [0.39, 2.51]	0.977
Healthcare utilisation							
Cardiovascular disease, *n*(%)	1 (0.7)	141	2 (1.5)	133	0.47 [0.04, 5.20]	0.43 [0.04, 4.83]	0.489
Respiratory disease, *n*(%)	4 (2.8)	141	3 (2.3)	133	1.26 [0.29, 5.55]	1.13 [0.25, 5.07]	0.874
Gastroenterology disease, *n*(%)	4 (2.8)	141	3 (2.3)	133	1.26 [0.29, 5.55]	1.23 [0.28, 5.52]	0.784
Renal disease, *n*(%)	0	141	0	133	NE	NE	NE
Gynaecological disease, *n*(%)	0	141	4 (3.0)	133	NE	NE	NE
Neurological disease, *n*(%)	1 (0.7)	141	3 (2.3)	133	0.31 [0.03, 3.01]	0.30 [0.03, 2.97]	0.305
Mental health problem, *n*(%)	1 (0.7)	141	3 (2.3)	133	0.31 [0.03, 3.02]	0.32 [0.03, 3.14]	0.328
Musculoskeletal disease, *n*(%)	1 (0.7)	141	2 (1.5)	133	0.47 [0.04, 5.20]	0.44 [0.04, 4.92]	0.501
Endocrinological problem, *n*(%)	3 (2.1)	141	5 (3.8)	133	0.57 [0.14, 2.34]	0.49 [0.12, 2.06]	0.327
Dermatological problem, *n*(%)	1 (0.7)	141	0	133	NE	NE	0.979
Other, *n*(%)	4 (2.8)	141	2 (1.5)	133	1.89 [0.35, 10.21]	1.82 [0.33, 9.95]	0.490
Health Related Quality of Life SF-36							
Physical component score	50.9 (8.6)	106	51.8 (7.1)	118	−0.95 [−3.02, 1.12]	−1.02 [−3.13, 1.08]	0.342
Mental component score	46.3 (8.9)	106	45.1 (8.8)	118	1.19 [−1.13, 3.54]	1.38 [−0.99, 3.76]	0.254
Physical functioning	84.4 (23.7)	106	85.9 (20.9)	119	−1.45 [−7.31, 4.41]	−2.20 [−8.13, 3.74]	0.467
Role physical	84.8 (30.5)	107	88.2 (26.0)	119	−3.42 [−10.83, 3.98]	−3.50 [−11.03, 4.03]	0.362
Bodily pain	71.7 (23.6)	107	72.8 (23.0)	119	−1.12 [−7.24, 4.99]	−0.43 [−6.62, 5.76]	0.898
General health	68.5 (20.8)	107	66.3 (17.8)	120	2.27 [−2.78, 7.31]	2.54 [−2.58, 7.67]	0.333
Vitality	56.5 (15.1)	107	54.4 (14.4)	120	2.10 [−1.76, 5.95]	2.33 [−1.59, 6.24]	0.244
Social functioning	80.6 (22.3)	107	81.5 (20.0)	120	−0.85 [−6.39, 4.68]	−0.94 [−6.57, 4.69]	0.745
Role emotional	85.1 (29.4)	107	85.9 (29.0)	118	−0.83 [−8.51, 6.86]	−0.03 [−7.82, 7.76]	0.996
Mental health	68.2 (13.5)	107	65.6 (12.6)	119	2.54 [−0.89, 5.96]	2.56 [−0.92, 6.04]	0.151
EPDS ≥12, n (%)	17 (18.3)	93	20 (16.9)	118	1.07 [0.60, 1.95]	1.12 [0.61, 2.05]	0.706
EPDS	6.9 (5.2)	93	7.4 (4.7)	118	−0.54 [−1.90,0.82]	−0.48 [−1.86,0.91]	0.505
Short form STAI ≥15, *n*(%)	39 (36.8)	106	54 (45.0)	120	0.82 [0.59, 1.13]	0.89 [0.64, 1.22]	0.464
Short form STAI	13.7 (2.2)	106	14.1 (2.2)	120	−0.49 [−1.06, 0.08]	−0.38 [−0.96, 0.19]	0.192

Data are mean (standard deviation, SD) unless otherwise indicated. No. = number of participants providing information for that outcome. Treatment effects are relative risks or mean differences and 95% confidence interval (CI). GA OGTT = gestational age at oral glucose tolerance test. HbA1c = glycated haemoglobin.

*Metabolic syndrome is defined as three or more of: hypertension; triglycerides >1.7 mmol/L; HDL-cholesterol <1.29 mmol/L; FPG ≥ 5.6 mmol/L; pre-diabetes or diabetes; obesity (BMI ≥ 30 kg/m^2^ [10,11].

†Prescriptions for the treatment of pre-diabetes included self-reported treatment with medication or lifestyle. Prescriptions for the treatment of type 2 diabetes included self-reported treatment with insulin or medication or lifestyle. SF-36 = Short-Form Health Related Quality of Life [12]. EPDS = Edinburgh Postnatal Depression Score [13]. STAI = Short-Form State-Trait Anxiety Inventory [14]. NE = Not estimable.

The primary outcome for the child of BMI z-score was similar between the tight and less tight glycaemic groups (mean z-score 0.83 SD 1.72 versus 0.75 SD 1.48; adjMD 0.12 (95% CI [−0.24, 0.48]; *P* = 0.498)) (Table 3) and remained similar after the prespecified additional adjustments for gestational age at birth (adjMD 0.10 (95% CI [−0.26, 0.46]; *P* = 0.573)), maternal ethnicity (adjMD 0.04 (95% CI [−0.31, 0.40]; *P* = 0.810)), use of pharmacological treatment for GDM (adjMD 0.06 (95% CI [−0.31, 0.42]; *P* = 0.751)), trial cluster (adjMD 0.12 (95% CI [−0.28, 0.51]; *P* = 0.565)), socioeconomic status (adjMD 0.16 (95% CI [−0.22, 0.54]; *P* = 0.406)) and neonatal hypoglycaemia (adjMD 0.14 (95% CI [−0.22, 0.50]; *P* = 0.439)) (S3 Table).

**Table 3:** Primary and secondary outcomes among the children at 4.5 Year Follow-Up.

	Tight glycaemic group	No. *n* = 162	Less tight glycaemic group	No. *n* = 151	Unadjusted treatment effect [95% CI]	Treatment effect adjusted for GA OGTT [95% CI]	*P* value
Primary outcome							
BMI z-score	0.83 (1.72)	157	0.75 (1.48)	149	0.08 [−0.28, 0.44]	0.12 [−0.24, 0.48]	0.498
Secondary outcomes							
Overweight/obese (BMI z-score >2), *n*(%)	24 (15.3)	157	20 (13.4)	149	1.14 [0.66, 1.98]	1.36 [0.79, 2.34]	0.268
Obese (BMI z-score >3), *n*(%)	13 (8.3)	157	11 (7.4)	149	1.12 [0.52, 2.43]	1.29 [0.59, 2.80]	0.521
BMI (kg/m^2^)	16.7 (3.1)	157	16.5 (2.4)	149	0.21 [−0.41, 0.83]	0.30 [−0.32, 0.91]	0.347
Height (cm)	107.8 (5.5)	157	106.0 (5.5)	149	1.79 [0.55, 3.04]	1.83 [0.58, 3.08]	0.004
Height z-score	0.5 (1.1)	157	0.1 (1.0)	149	0.33 [0.09, 0.57]	0.35 [0.11, 0.59]	0.005
Short stature (height z-score < −2), *n*(%)	0	157	3 (2.0)	149	NE	NE	NE
Weight (kg)	19.6 (4.9)	157	18.7 (3.8)	149	0.92 [−0.06, 1.90]	1.04 [0.06, 2.02]	0.037
Weight z-score	0.8 (1.5)	157	0.6 (1.3)	149	0.24 [−0.07, 0.55]	0.28 [−0.03, 0.59]	0.079
Underweight (z-score < −2), *n*(%)	1 (0.6)	157	0 (0)	149	NE	NE	NE
Normal weight (z-score-2–2), *n*(%)	134 (85.4)	157	133 (89.3)	149	0.96 [0.88, 1.04]	0.97 [0.90, 1.06]	0.516
Overweight (z-score >2–3), *n*(%)	10 (6.4)	157	9 (6.0)	149	1.05 [0.44, 2.53]	1.18 [0.49, 2.83]	0.712
Obese (z-score >3), *n*(%)	12 (7.6)	157	7 (4.7)	149	1.63 [0.66, 4.04]	1.87 [0.75, 4.66]	0.179
Any neurosensory disability, *n*(%)^*^	14 (9.8)	143	8 (6.0)	133	1.63 [0.70, 3.77]	1.62 [0.70, 3.78]	0.259
-Cerebral palsy, *n*(%)	0 (0)	142	0 (0)	132	NE	NE	NE
-Blindness, *n*(%)	0 (0)	162	0 (0)	151	NE	NE	NE
-Deafness (difficulty hearing but does not need aids), *n*(%)	0 (0)	141	3 (2.3)	130	NE	NE	NE
-Developmental delay (language, motor or cognitive), *n*(%)	14 (9.9)	141	7 (5.4)	129	1.83 [0.76, 4.41]	1.83 [0.76, 4.43]	0.180
Coordination impairment, *n*(%)	2 (1.4)	143	2 (1.5)	131	0.92 [0.13, 6.47]	0.89 [0.12, 6.42]	0.911
Other visual problems, *n*(%)	1 (0.7)	143	0	133	NE	NE	NE
Other hearing problems, *n*(%)	0	141	3 (2.3)	130	NE	NE	NE
Attending special care programme, *n*(%)	5 (3.6)	140	3 (2.3)	131	1.56 [0.38, 6.40]	2.09 [0.50, 8.78]	0.316
-Vision, *n*(%)	0	140	0	131	NE	NE	NE
-Developmental delay, *n*(%)	3 (2.1)	140	1 (0.8)	131	2.80 [0.29, 26.83]	3.14 [0.32, 30.48]	0.322
-Hearing, *n*(%)	0	140	2 (1.5)	131	NE	NE	NE
-Behavioural problems, *n*(%)	0	140	0	131	NE	NE	NE
-Cerebral palsy, *n*(%)	0	140	0	131	NE	NE	NE
-Other, *n*(%)	2 (1.4)	140	0	131	NE	NE	NE
Supportive care in last 2 years, *n*(%)	15 (10.7)	140	9 (6.9)	130	1.55 [0.70, 3.43]	1.56 [0.70, 3.48]	0.305
-Speech pathology, *n*(%)	11 (7.9)	140	6 (4.6)	130	1.71 [0.65, 4.52]	1.74 [0.65, 4.62]	0.267
-Psychological assessment, *n*(%)	0	140	2 (1.5)	130	NE	NE	NE
-Physiotherapy, *n*(%)	0	140	1 (0.8)	130	NE	NE	NE
-Occupational therapy, *n*(%)	4 (2.9)	140	0	130	NE	NE	NE
-Other, *n*(%)	0	140	0	130	NE	NE	NE
Little Developmental Coordination Disorder Questionnaire							
Coordination difficulty, *n*(%)^†^	31 (28.4)	109	21 (17.8)	118	1.60 [0.98, 2.61]	1.66 [1.01, 2.73]	0.044
Little DCDQ overall score	68.0 (9.7)	109	70.8 (6.2)	118	−2.74 [−4.85, −0.62]	−2.84 [−4.99, −0.69]	0.010
-Gross motor factor subscale score	40.6 (6.3)	109	42.1 (4.2)	118	−1.48 [−2.87, −0.10]	−1.44 [−2.84, −0.03]	0.046
-Fine motor factor subscale score	27.4 (4.2)	109	28.6 (2.4)	118	−1.25 [−2.14, −0.36]	−1.41 [−2.32, −0.51]	0.002
Strengths and Difficulties Questionnaire							
Total difficulty score (score ≥14), *n*(%)	16 (14.3)	112	10 (8.3)	121	1.73 [0.82, 3.66]	1.77 [0.83, 3.78]	0.138
Total difficulties score	8.4 (5.1)	112	6.8 (4.5)	121	1.57 [0.34, 2.80]	1.75 [0.51, 3.00]	0.006
Emotional symptoms, *n*(%)	14 (12.5)	112	10 (8.3)	121	1.51 [0.70, 3.28]	1.60 [0.73, 3.49]	0.239
Conduct problems, *n*(%)	26 (23.2)	112	20 (16.5)	121	1.40 [0.83, 2.38]	1.44 [0.85, 2.44]	0.178
Hyperactivity, *n*(%)	19 (17.0)	112	11 (9.1)	121	1.87 [0.93, 3.76]	1.87 [0.92, 3.79]	0.085
Peer problems, *n*(%)	31 (27.7)	112	23 (19.0)	121	1.46 [0.90, 2.35]	1.61 [1.00, 2.59]	0.048
Prosocial behaviour, *n*(%)	8 (7.1)	112	6 (5.0)	121	1.44 [0.51,4.04]	1.38 [0.49,3.94]	0.540
Social Communication Questionnaire							
Likely on the autism spectrum, n (%)^‡^	10 (9.3)	108	3 (2.6)	117	3.61 [1.01, 12.86]	3.67 [1.02, 13.23]	0.047
Total score	7.3 (5.1)	108	5.6 (3.8)	117	1.69 [0.52, 2.88]	1.84 [0.65, 3.03]	0.003
Child Eating Behaviour Questionnaire							
Food responsiveness	2.0 (0.7)	112	1.9 (0.6)	118	0.09 [−0.08, 0.25]	0.12 [−0.05, 0.28]	0.175
Enjoyment of food	3.0 (0.7)	112	3.0 (0.6)	118	−0.03 [−0.21, 0.15]	−0.03 [−0.21, 0.15]	0.718
Emotional overeating	1.4 (0.6)	112	1.4 (0.4)	118	0.02 [−0.10, 0.15]	0.03 [−0.10, 0.16]	0.628
Desire to drink	2.1 (0.9)	112	2.0 (0.7)	118	0.05 [−0.15, 0.25]	0.08 [−0.12, 0.29]	0.419
Satiety responsiveness	2.3 (0.6)	112	2.3 (0.5)	118	−0.007 [−0.14, 0.13]	−0.01 [−0.15, 0.12]	0.827
Slowness in eating	2.4 (0.6)	112	2.5 (0.6)	118	−0.03 [−0.18, 0.12]	−0.03 [−0.19, 0.12]	0.700
Emotional undereating	2.1 (0.7)	112	2.0 (0.6)	118	0.05 [−0.11, 0.21]	0.05 [−0.11, 0.21]	0.547
Food fussiness	2.6 (0.7)	112	2.4 (0.7)	118	0.17 [−0.005, 0.34]	0.20 [0.02, 0.37]	0.029
Child Health Questionnaire PF50							
Low physical functioning, *n*(%)^§^	7 (6.6)	106	5 (4.4)	113	1.49 [0.49, 4.59]	1.59 [0.51, 4.91]	0.422
Low psychosocial functioning, *n*(%)^§^	8 (7.6)	106	3 (2.7)	113	2.84 [0.77, 10.51]	2.80 [0.75, 10.43]	0.124
Physical functioning summary score	54.3 (7.2)	106	54.7 (8.6)	113	−0.45 [−2.56, 1.66]	−0.56 [−2.70, 1.57]	0.603
Psychosocial functioning summary score	51.8 (8.9)	106	55.0 (5.9)	113	−3.17 [−5.17, −1.17]	−3.10 [−5.13, −1.08]	0.003

Data are mean (standard deviation, SD) unless otherwise indicated. No. = number of participants providing information for that outcome. Treatment effects are relative risks or mean differences and 95% confidence interval (CI). GA OGTT = gestational age at oral glucose tolerance test. Body size determined using WHO charts [15].

*Composite of neurosensory disability defined as any of cerebral palsy, blindness, deafness, or developmental delay.

†Coordination difficulty is defined as <67 for boys and <68 for girls on the Little Developmental Coordination Disorder Questionnaire [16].

‡Likely on the autism spectrum, defined as ≥15 on the Social Communication Questionnaire [19].

§Low physical and psychosocial functioning is defined as a score <40 on the Child Health Questionnaire PF50 [22]. NE = Not estimable.

In post-hoc analysis adjustment for time and the effect of clustering within each step-wedge produced similar results for the primary outcomes (S4 Table).

There was no difference between glycaemic groups for most of the secondary maternal outcomes including the diagnosis of diabetes or pre-diabetes, medication use for diabetes, body size, total cholesterol, high-density lipoprotein (HDL), low-density lipoprotein (LDL), hypertension, diagnosis of metabolic syndrome, further pregnancies, healthcare utilisation, health related quality of life, and vulnerability to depression and anxiety, although triglycerides were slightly higher in the tight glycaemic group (mean 1.8 mmol/L SD 0.8 versus mean 1.6 mmol/L SD 0.7; adjMD 0.25 (95% CI [0.01, 0.49]; *P* = 0.039)) (Table 2).

For the secondary child outcomes, children in the tight glycaemic target group weighed more (19.6 kg SD 4.9 versus 18.7 kg SD 3.8; adjMD 1.04 (95% CI [0.06, 2.02]; *P* = 0.037)) although their weight z-score was not different between groups. Children in the tight glycaemic target group were taller than children in the less tight group (107.8 cm SD 5.5 versus 106.0 cm SD 5.5; adjMD 1.83 (95% CI [0.58, 3.08]; *P* = 0.004)) and their height z-score was greater (0.5 SD 1.1 versus 0.1 SD 1.0; adjMD 0.35 (95% CI [0.11, 0.59]; *P* = 0.005)) (Table 3). The differences in child height and height z-score persisted after additional adjustment in the prespecified exploratory analyses (S3 Table).

Children in the tight glycaemic group compared with children in the less tight glycaemic group were more likely to have a coordination difficulty (31/109, 28.4% versus 21/118, 17.8%; adjusted relative risk (adjRR) 1.66 (95% CI [1.01, 2.73]; *P* = 0.044)), and to be likely on the autism spectrum (10/108, 9.3% versus 3/117, 2.6%; adjRR 3.67 (95% CI [1.02, 13.23]; *P* = 0.047)). Children from the tight glycaemic group compared to the less tight group had higher mean difficulties scores on the SDQ (mean score 8.4 SD 5.1 versus mean score 6.8 SD 4.5; adjMD 1.75 (95% CI [0.51, 3.00]; *P* = 0.006)), although the risk of behavioural difficulties (SDQ score ≥14) was not significantly higher (16/112, 14.3% versus 10/121, 8.3%; adjRR 1.77 (95% CI [0.83, 3.78]; *P* = 0.138)) (Table 3). In the prespecified exploratory analyses, there was decreased precision but the direction of effect remained the same, with overall worse motor and behavioural outcomes in the tight glycaemic target group (S3 Table). There were no differences between glycaemic groups for child neurological status and for most components of the child eating behaviour and functional health and wellbeing questionnaires. Children in the tight glycaemic group scored higher in food fussiness (mean score 2.6 SD 0.7 versus 2.4 SD 0.7; adjMD 0.20 (95% CI [0.02, 0.37]; *P* = 0.029)) and had worse (lower) psychosocial functioning summary scale scores (mean score 51.8 SD 8.9 versus 55.0 SD 5.9; adjMD −3.10 (95% CI [−5.13, −1.08]; *P* = 0.003)) (Table 3).

In the post-hoc sensitivity analysis comparing the continuous child neurodevelopmental outcomes the nonparametric approach generally agreed with the prespecified parametric approach with similar directions of effect, however the physical functioning summary score of the Child Health Questionnaire was only significant in the nonparametric analysis (S5 Table).

## Discussion

This study is a prospective longitudinal follow-up of women with GDM and their children who participated in a randomised trial of tight or less tight targets for glycaemic control and compared their health outcomes 4.5 years after birth. There is limited research on whether different glycaemic targets for the treatment of GDM influence the later health of mothers and their children.

The primary outcome of maternal HbA1c was not different between glycaemic target groups. Tight glycaemic targets did not result in improvement in any of the secondary maternal outcomes assessed, nor did they result in any adverse effects. Thus, these two different glycaemic targets recommended for treatment of GDM do not appear to influence later maternal health.

The primary outcome of child BMI z-score was also not different between the tight and less tight glycaemic groups. While there was no difference in birthweight between groups, we had hypothesised that women in the tight glycaemic target group may have adopted a greater focus on lifestyle modification during pregnancy to achieve their targets, which may have continued postpartum in household lifestyle modification, resulting in their children having a lower BMI z-score at follow-up. In the Cochrane review on glycaemic targets during pregnancy, none of the included trials reported on BMI in later childhood [5]. Child follow-up from other studies of women with GDM who have been recommended to use different glycaemic targets is needed to further evaluate any effects on later childhood growth.

Children in the tight glycaemic target group had worse behavioural outcomes when compared with their peers in the less tight target group. Overall, 5.8% of children had a score indicating they were likely to be on the autism spectrum which is higher than the reported rate of autism spectrum disorder in the general New Zealand population of 1.3–1.7% for children aged 4–5 years [29]. However, the proportion of children with emotional and behavioural difficulties (11.2% with SDQ scores ≥14) was similar when compared to the 2018 Ministry of Health New Zealand health survey (10.2% with SDQ scores >15 [30]).

Adverse neurodevelopmental outcomes in the offspring of women in the tight glycaemic target group compared to the less tight target group were unexpected. Adverse neurocognitive and behavioural outcomes have been reported previously in children exposed to GDM. A recent meta-analysis included cohort, cross-sectional, and case control studies and reported on the risk of autism spectrum disorder (1,877,164 participants in 18 studies) and attention deficit hyperactivity disorder (992,310 participants in 15 studies) in children of women with GDM [31]. The pooled odds ratio for the risk of autism spectrum disorder following GDM exposure was 1.42 (95% CI [1.22, 1.65]) and for attention deficit hyperactivity disorder was 1.01 (95% CI [0.79, 1.28]). Our findings suggest that recommendations to use tight glycaemic targets may contribute to an increased risk of autism spectrum disorder with GDM exposure and further assessment is warranted in later childhood.

Children in the tight glycaemic group had worse motor outcomes than children in the less tight glycaemic group. The overall frequency of a coordination difficulty in our study was higher than in the general New Zealand population (23% versus 10%) [32], consistent with previous reports that motor outcomes are worse in children of mothers with GDM than in children not exposed to GDM [33].

We found that children in the tight glycaemic group were slightly taller and heavier than children in the less tight group. In a clinical trial, treatment of mild GDM compared with no treatment did not affect infant linear growth [34]. However, in a longitudinal study from the UK involving 9,901 children aged 5 years, 730 of whom were born to mothers with GDM, height z-scores were increased in children whose mothers were treated with insulin (adjMD 0.22 (95% CI [0.075, 0.36]; *P* = 0.003)) or metformin (adjMD 0.35 (95% CI [0.027, 0.68]; *P* = 0.034)), compared to children of mothers without GDM [35]. This finding was not seen in children whose mothers were treated with lifestyle changes alone for their GDM (adjMD 0.04 (95% CI [−0.22, 0.14]; *P* = 0.67)).

Our study has a number of strengths. This follow-up study describes the longer-term outcomes in mothers and their children who participated in the New Zealand-wide TARGET trial on the use of different glycaemic targets for GDM during pregnancy. Tight glycaemic targets were recommended in New Zealand at the time of the original trial, based on the New Zealand Ministry of Health guidance [1]. This study gives important feedback on this recommended change in clinical practice about the effects on later health for mothers and their children. There have only been four randomised controlled trials comparing different glycaemic targets in pregnancy and only our study has, to date, reported on the follow-up of mothers and their children beyond birth.

Our study has some limitations. The TARGET trial included a waiver of consent and only participants who had consented were considered for eligibility in this follow-up study. Our post-hoc follow-up study assessed many secondary health outcomes considered of importance for the mother and her child so increasing the risk of a type 1 error, since no adjustment for multiplicity was perfomed. In addition, for some of the outcomes, not all eligible participants were able to contribute data. Screening questionnaires were utilised for child motor and behavioural outcomes and we acknowledge that a health professional assessment would be required to confirm or exclude a diagnosis of autism spectrum disorder or a developmental coordination disorder.

This post-hoc follow-up study of tight compared to less tight glycaemic targets in women with GDM did not result in lower maternal HbA1c or lower child BMI z-scores 4.5 years later, and may be associated with adverse child motor and behavioural outcomes. The potential adverse child behavioural and motor findings observed for children of mothers allocated to the tight glycaemic targets highlight the need for other studies to report on childhood health following the use of different targets for glycaemic control in women with GDM.

## Supporting information

S1 Table Baseline TARGET trial entry characteristics of mothers and children who were eligible for follow-up at 4.5 years and participated or did not participate. (DOCX)

S2 Table Maternal outcomes analysed with prespecified additional adjustments for gestational age at birth, maternal ethnicity, pharmacological treatment of GDM, trial cluster and, socioeconomic status. (DOCX)

S3 Table Child outcomes analysed with prespecified additional adjustments for gestational age at birth, maternal ethnicity, pharmacological treatment of GDM, trial cluster, socioeconomic status, and neonatal hypoglycaemia. (DOCX)

S4 Table Post-hoc sensitivity analysis for primary maternal and child outcomes adjusted for stepped-wedge trial design. (DOCX)

S5 Table Post-hoc sensitivity analysis: child neurodevelopmental outcomes using a nonparametric approach (median difference) compared with a parametric (mean difference) approach. (DOCX)

S1 Fig Box and whisker plots of primary and key child continuous neurodevelopmental outcomes. (DOCX)

S1 File The TARGET 4.5 Year Follow-Up Study Protocol. (DOCX)

S2 File The TARGET 4.5 Year Follow-Up Study Statistical Analysis Plan. (DOCX)

S1 Checklist CONSORT checklist. This checklist is licensed under the Creative Commons Attribution 4.0 International License (CC BY 4.0; https://creativecommons.org/licenses/by/4.0/) https://doi.org/10.1371/journal.pmed.1004662.s001.
(DOCX)

## Abbreviations

adjMD: adjusted mean difference
adjRR: adjusted relative risk
B4SC: before school check
BMI: body mass index
CEBQ: Child Eating Behaviour Questionnaire
CHQ: Child Health Questionnaire
CI: confidence interval
CONSORT: Consolidated Standards of Reporting Trials
EPDS: Edinburgh Postnatal Depression Score
FPG: fasting plasma glucose
GA: gestational age
GDM: gestational diabetes mellitus
GLMM: generalised linear mixed-effects model
HAPO: Hyperglycemia and Adverse Pregnancy Outcomes
HbA1c: glycated haemoglobin
HDL: high-density lipoprotein
LDL: low-density lipoprotein
Little DCDQ: Little Developmental Coordination Disorder Questionnaire
OGTT: oral glucose tolerance test
RR: relative risk
SCQ: Social Communication Questionnaire
SD: standard deviation
SDQ: Strengths and Difficulties Questionnaire
STAI: State-Trait Anxiety Inventory
