# Supplementary material for: Mother and child health 4.5 years after gestational diabetes mellitus managed using tight or less tight targets for glycaemic control: Post-hoc follow-up study of the TARGET trial
Source: PLoS Med. 2026 Feb 3;23(2):e1004635. doi: 10.1371/journal.pmed.1004635 (PMC12867249; doi:10.1371/journal.pmed.1004635)
Supplement: S1 Table — (DOCX) [file pmed.1004635.s001.docx]

**S1 Table: Baseline TARGET Trial entry characteristics of mothers and children who were eligible for follow-up at 4.5 years and participated or did not participate.**

| Mothers | Mothers who participated | No. n=315 | Mothers who did not participate | No. n=112 |
| --- | --- | --- | --- | --- |
| Age (years) | 33.2 (4.9) | 315 | 32.3 (5.1) | 111 |
| Primiparity, n (%) | 125 (39.7) | 315 | 51 (45.5) | 112 |
| Gestational age at entry to TARGET trial (weeks)^*^ | 27.7 (26.1, 28.9) | 315 | 27.4 (26.1, 28.8) | 112 |
| BMI (kg/m^2^) | 32.4 (7.2) | 312 | 32.6 (6.7) | 112 |
| Underweight (<18.5), n (%) | 0 (0) | 312 | 0 (0) | 112 |
| Normal weight (18.5-24.9), n (%) | 29 (9.3) | 312 | 14 (12.5) | 112 |
| Overweight (25.0-29.9), n (%) | 103 (33.0) | 312 | 32 (28.6) | 112 |
| Obese (≥30.0), n (%) | 180 (57.7) | 312 | 66 (58.9) | 112 |
| Smoking at trial entry, n (%) | 22 (7.0) | 315 | 10 (8.9) | 112 |
| Previous history of GDM prior to index pregnancy, n (%) | 60 (19.2) | 315 | 22 (19.6) | 112 |
| Prioritised ethnicity† |  |  |  |  |
| Māori, n (%) | 25 (7.9) | 315 | 15 (13.4) | 112 |
| Pacific, n (%) | 40 (12.7) | 315 | 16 (14.3) | 112 |
| New Zealand European, n (%) | 152 (48.3) | 315 | 31 (27.7) | 112 |
| Asian, n (%) | 92 (29.2) | 315 | 48 (42.9) | 112 |
| Other, n (%) | 6 (1.9) | 315 | 2 (1.8) | 112 |
| New Zealand Deprivation Category^‡^ |  |  |  |  |
| 1-2, n (%) – least deprived | 45 (15.0) | 300 | 12 (11.0) | 109 |
| 3-4, n (%) | 56 (18.7) | 300 | 7 (6.4) | 109 |
| 5-6, n (%) | 44 (14.7) | 300 | 16 (14.7) | 109 |
| 7-8, n (%) | 66 (22.0) | 300 | 23 (21.0) | 109 |
| 9-10, n (%) – most deprived | 89 (29.7) | 300 | 51 (46.8) | 109 |
| OGTT plasma glucose concentrations |  |  |  |  |
| Fasting glucose (mmol/L)^*^ | 5.0 (4.5, 5.7) | 315 | 4.9 (4.4, 5.7) | 112 |
| 2 hour glucose (mmol/L)^*^ | 9.5 (9.0, 10.2) | 315 | 9.4 (9.0, 10.1) | 112 |
| Children | Children who participated | No. n=313 | Children who did not participate | No. n=114 |
| Gestational age at birth, (weeks)^*^ | 38.7 (38.0, 39.3) | 313 | 38.6 (37.9, 39.1) | 114 |
| Male sex, n (%) | 144 (46.0) | 313 | 68 (60.2) | 113 |
| Birthweight (g) | 3308 (527) | 313 | 3316 (579) | 114 |
| LGA, n (%) | 39 (12.5) | 313 | 17 (14.9) | 114 |
| SGA, n (%) | 18 (5.8) | 313 | 12 (10.5) | 114 |
| Neonatal hypoglycaemia, n (%) | 86 (27.5) | 313 | 34 (30.4) | 114 |
| Prioritised ethnicity^†^ |  |  |  |  |
| Māori, n (%) | 25 (8.0) | 313 | 15 (13.2) | 114 |
| Pacific, n (%) | 40 (12.8) | 313 | 16 (14.0) | 114 |
| New Zealand European, n (%) | 127 (40.6) | 313 | 31 (27.2) | 114 |
| Asian, n (%) | 90 (28.8) | 313 | 49 (43.0) | 114 |
| Other, n (%) | 31 (9.9) | 313 | 2 (1.8) | 114 |

Data are mean (SD) unless otherwise indicated. No. = number of participants providing information for that outcome. BMI = Body Mass Index. OGTT = oral glucose tolerance test. LGA = Large for gestational age. SGA = Small for gestational age. ^*^median (Q1, Q3). ^†^Ethnicity was determined by self-report, according to the New Zealand Ministry of Social Development and prioritised for analysis [23]. ^‡^New Zealand Deprivation Category determined using the New Zealand Deprivation Index (NZDEP) [24].
