# Supplementary material for: Mother and child health 4.5 years after gestational diabetes mellitus managed using tight or less tight targets for glycaemic control: Post-hoc follow-up study of the TARGET trial
Source: PLoS Med. 2026 Feb 3;23(2):e1004635. doi: 10.1371/journal.pmed.1004635 (PMC12867249; doi:10.1371/journal.pmed.1004635)
Supplement: S2 Table — (DOCX) [file pmed.1004635.s002.docx]

**S2 Table: Maternal outcomes analysed with pre-specified additional adjustments for gestational age at birth, maternal ethnicity, pharmacological treatment of GDM, trial cluster and socioeconomic status.**

|  | GA at birth (pre-term or term) | P value | Maternal ethnicity | P value | Treatment* of GDM | P value | Trial cluster^†^ | P value | Socioeconomic status | P value |
| --- | --- | --- | --- | --- | --- | --- | --- | --- | --- | --- |
| Primary Outcome |  |  |  |  |  |  |  |  |  |  |
| Maternal HbA1c (mmol/mol) | 2.03 [-0.40,4.45] | 0.102 | 0.62 [-1.73,2.96] | 0.607 | 1.41 [-1.00,3.83] | 0.249 | 0.66 [-2.00,3.33] | 0.623 | 1.60 [-0.96,4.15] | 0.220 |
| Maternal HbA1c (%) | 0.19 [-0.04,0.41] | 0.102 | 0.06 [-0.16,0.27] | 0.607 | 0.13 [-0.09,0.35] | 0.249 | 0.06 [-0.18,0.30] | 0.623 | 0.15 [-0.09,0.38] | 0.220 |
| Secondary Outcomes |  |  |  |  |  |  |  |  |  |  |
| Any of Pre-diabetes or Diabetes | 1.00 [0.67,1.50] | 0.982 | 0.84 [0.61,1.15] | 0.275 | 0.92 [0.62,1.37] | 0.500 | 0.84 [0.60,1.17] | 0.297 | 0.86 [0.63,1.18] | 0.353 |
| Pre-diabetes | 1.00 [0.67,1.50] | 0.998 | 0.85 [0.57,1.27] | 0.431 | 0.92 [0.62,1.37] | 0.698 | 0.81 [0.53,1.23] | 0.327 | 0.87 [0.59,1.30] | 0.503 |
| Type 2 diabetes | 1.03 [0.44,2.41] | 0.941 | 0.88 [0.37,2.06] | 0.765 | 0.89 [0.38,2.07] | 0.783 | 0.80 [0.34,1.88] | 0.606 | 0.87 [0.38,2.01] | 0.751 |
| Height (cm) | 0.06 [-1.42,1.55] | 0.932 | 0.44 [-0.99,1.88] | 0.543 | -0.06 [-1.57,1.44] | 0.937 | 0.69 [-0.94,2.33] | 0.405 | 0.10 [-1.45,1.64] | 0.901 |
| Weight (kg) | -0.45 [-5.90,5.00] | 0.870 | -1.22 [-6.18,3.74] | 0.630 | -2.35 [-7.72,3.02] | 0.391 | -1.83 [-7.85,4.18] | 0.549 | -0.70 [-6.18,4.78] | 0.801 |
| BMI (kg/m^2^) | -0.15 [-1.99,1.70] | 0.874 | -0.53 [-2.24,1.18] | 0.544 | -0.80 [-2.61,1.02] | 0.388 | -0.91 [-2.93,1.12] | 0.377 | -0.25 [-2.11,1.61] | 0.794 |
| Metabolic syndrome^‡^ | 1.01 [0.65,1.57] | 0.961 | 0.88 [0.57,1.36] | 0.558 | 0.94 [0.61,1.46] | 0.789 | 0.83 [0.52,1.34] | 0.446 | 0.93 [0.60,1.45] | 0.755 |

Treatment effects as relative risks or mean differences and 95% confidence intervals. GA = gestational age. ^*^Pharmacological treatment using metformin or insulin or both. ^†^Trial cluster refers to hospital pairs. Socioeconomic status, determined using the NZ Deprivation Index (NZDEP) [24]. BMI = Body Mass Index. ^‡^Metabolic syndrome defined as three or more of: hypertension; triglycerides >1.7mmol/L; High-density lipoprotein-cholesterol <1.29mmol/L; Fasting plasma glucose >5.6mmol/L; pre-diabetes or diabetes; obesity (BMI >30kg/m^2^) [10, 11].
