# Supplementary material for: Mother and child health 4.5 years after gestational diabetes mellitus managed using tight or less tight targets for glycaemic control: Post-hoc follow-up study of the TARGET trial
Source: PLoS Med. 2026 Feb 3;23(2):e1004635. doi: 10.1371/journal.pmed.1004635 (PMC12867249; doi:10.1371/journal.pmed.1004635)
Supplement: S3 Table — (DOCX) [file pmed.1004635.s003.docx]

**S3 Table: Child outcomes analysed with pre-specified additional adjustments for gestational age at birth, maternal ethnicity, pharmacological treatment of GDM, trial cluster, socioeconomic status and neonatal hypoglycaemia.**

|  | GA at birth (term or pre-term) | P value | Maternal ethnicity | P value | Treatment* of GDM | P value | Trial cluster^†^ | P value | Socioeconomic status | P value | Neonatal hypo-glycaemia | P value |
| --- | --- | --- | --- | --- | --- | --- | --- | --- | --- | --- | --- | --- |
| Primary outcome |  |  |  |  |  |  |  |  |  |  |  |  |
| BMI z -score | 0.10 [-0.26,0.46] | 0.573 | 0.04 [-0.31,0.40] | 0.810 | 0.06 [-0.31,0.42] | 0.751 | 0.12 [-0.28,0.51] | 0.565 | 0.16 [-0.22,0.54] | 0.406 | 0.14 [-0.22,0.50] | 0.439 |
| Secondary outcomes |  |  |  |  |  |  |  |  |  |  |  |  |
| Overweight/obese (BMI z-score >2) | 1.28 [0.75,2.18] | 0.362 | 1.23 [0.75,2.04] | 0.412 | 1.21 [0.69,2.09] | 0.506 | 1.15 [0.65,2.05] | 0.633 | 1.48 [0.84,2.60] | 0.176 | 1.39 [0.80,2.40] | 0.237 |
| Obese (BMI z-score >3) | 1.18 [0.55,2.54] | 0.667 | 1.04 [0.51,2.15] | 0.910 | 1.04 [0.48,2.24] | 0.919 | 1.00 [0.44,2.27] | 0.999 | 1.42 [0.63,3.20] | 0.391 | 1.33 [0.61,2.91] | 0.468 |
| BMI (kg/m^2^) | 0.25 [-0.36,0.87] | 0.417 | 0.11 [-0.50,0.71] | 0.724 | 0.17 [-0.45,0.80] | 0.583 | 0.25 [-0.43,0.93] | 0.466 | 0.34 [-0.30,0.99] | 0.298 | 0.32 [-0.30,0.94] | 0.311 |
| Height (cm) | 1.79 [0.54,3.05] | 0.005 | 1.45 [0.20,2.69] | 0.023 | 1.76 [0.49,3.04] | 0.007 | 1.60 [0.24,2.96] | 0.022 | 1.80 [0.50,3.10] | 0.007 | 1.85 [0.60,3.11] | 0.004 |
| Height z-score | 0.34 [0.10,0.58] | 0.006 | 0.27 [0.03,0.51] | 0.025 | 0.34 [0.09,0.58] | 0.007 | 0.26 [-0.005,0.52] | 0.050 | 0.33 [0.08,0.58] | 0.010 | 0.35 [0.11,0.60] | 0.004 |
| Short stature (height z-score <-2) | NE |  | NE |  | NE |  | NE |  | NE |  | NE |  |
| Weight (kg) | 0.97 [-0.006,1.95] | 0.051 | 0.65 [-0.29,1.59] | 0.175 | 0.86 [-0.13,1.85] | 0.088 | 0.89 [-0.18,1.95] | 0.104 | 1.08 [0.06,2.09] | 0.037 | 1.07 [0.09,2.06] | 0.032 |
| Weight z-score | 0.26 [-0.05,0.57] | 0.102 | 0.17 [-0.13,0.47] | 0.252 | 0.23 [-0.09,0.54] | 0.160 | 0.21 [-0.13,0.55] | 0.218 | 0.29 [-0.03,0.61] | 0.079 | 0.29 [-0.02,0.60] | 0.066 |
| Underweight (z-score <-2) | NE |  | NE |  | NE |  | NE |  | NE |  | NE |  |
| Normal weight (z-score -2 to 2) | 0.97 [0.90,1.05] | 0.444 | 0.99 [0.81,1.21] | 0.925 | 0.97 [0.90,1.05] | 0.444 | 0.99 [0.88,1.11] | 0.812 | 0.92 [0.82,1.03] | 0.158 | 0.96 [0.87,1.05] | 0.373 |
| Overweight (z-score >2 to 3) | 1.10 [0.46,2.63] | 0.836 | 1.02 [0.42,2.46] | 0.974 | 1.05 [0.43,2.56] | 0.917 | 1.19 [0.46,3.08] | 0.726 | 1.21 [0.49,2.97] | 0.675 | 1.15 [0.48,2.77] | 0.752 |
| Obese (z-score >3) | 1.69 [0.69,4.18] | 0.252 | 1.47 [0.61,3.54] | 0.387 | 1.55 [0.62,3.87] | 0.347 | 1.47 [0.56,3.87] | 0.439 | 2.10 [0.80,5.53] | 0.133 | 2.07 [0.83,5.16] | 0.119 |
| Co-ordination difficulty^‡^ | 1.65 [1.00,2.71] | 0.049 | 1.63 [0.98,2.70] | 0.058 | 1.65 [1.00,2.70] | 0.049 | 1.53 [0.89,2.62] | 0.124 | 1.70 [1.02,2.83] | 0.043 | 1.64 [0.99,2.69] | 0.053 |
| Little DCDQ overall score | -2.75 [-4.91,-0.59] | 0.013 | -2.76 [-4.94,-0.58] | 0.013 | -2.88 [-5.08, 0.68] | 0.011 | -2.68 [-5.03,-0.32] | 0.026 | -2.80 [-5.08,-0.51] | 0.017 | -2.88 [-5.04,-0.72] | 0.009 |
| Total difficulty score (score ≥14) | 1.66 [0.77,3.59] | 0.192 | 1.58 [0.72,3.45] | 0.251 | 1.70 [0.79,3.68] | 0.174 | 1.59 [0.70,3.62] | 0.268 | 1.59 [0.75,3.35] | 0.221 | 1.76 [0.82,3.76] | 0.144 |
| Total difficulties score§ | 1.70 [0.45,2.95] | 0.008 | 1.46 [0.19,2.73] | 0.024 | 1.42 [0.16,2.67] | 0.027 | 1.49 [0.12,2.87] | 0.034 | 1.50 [0.20,2.79] | 0.024 | 1.75 [0.50,3.00] | 0.006 |
| Likely on the autism spectrum (score ≥15)^\|\|^ | 3.63 [1.01,13.08] | 0.049 | 2.90 [0.80,10.52] | 0.105 | 3.28 [0.90,11.92] | 0.072 | 2.08 [0.56,7.70] | 0.269 | 3.06 [0.86,10.88] | 0.083 | 3.72 [1.03,13.38] | 0.044 |
| SCQ Total score | 1.81 [0.61,3.00] | 0.003 | 1.36 [0.18,2.54] | 0.025 | 1.52 [0.31,2.73] | 0.014 | 1.44 [0.12,2.75] | 0.032 | 1.70 [0.48,2.92] | 0.007 | 1.80 [0.61,3.00] | 0.003 |
| Food responsiveness | 0.11 [-0.06,0.28] | 0.190 | 0.06 [-0.11,0.23] | 0.482 | 0.07 [-0.10,0.24] | 0.447 | 0.13 [-0.06,0.31] | 0.174 | 0.10 -0.08,0.28] | 0.260 | 0.11 [-0.05,0.28] | 0.185 |
| Enjoyment of food | -0.03 [-0.22,0.15] | 0.715 | -0.04 [-0.22,0.15] | 0.706 | -0.01[-0.20,0.17] | 0.907 | -0.02 [-0.22,0.18] | 0.822 | -0.06 [-0.25,0.12] | 0.512 | -0.04 [-0.22,0.15] | 0.700 |
| Emotional overeating | 0.03 [-0.10,0.15] | 0.670 | 0.003 [-0.13,0.13] | 0.962 | 0.02 [-0.11,0.15] | 0.789 | 0.04 [-0.10,0.18] | 0.597 | 0.02 [-0.12,0.15] | 0.797 | 0.03 [-0.10,0.15] | 0.667 |
| Desire to drink | 0.08 [-0.12,0.29] | 0.422 | -0.02 [-0.22,0.17] | 0.810 | 0.03 [-0.18,0.23] | 0.783 | -0.05 [-0.27,0.17] | 0.669 | 0.05 [-0.16,0.26] | 0.637 | 0.08 [-0.13,0.28] | 0.453 |
| Satiety responsiveness | -0.007 [-0.14,0.13] | 0.914 | -0.02 [-0.16,0.12] | 0.775 | -0.01 [-0.15,0.13] | 0..858 | -0.01 [-0.16,0.14] | 0.882 | -0.01 [-0.15,0.13] | 0.848 | -0.01 [-0.15,0.12] | 0.850 |
| Slowness in eating | -0.03 [0.19,0.12] | 0.685 | -0.07 [-0.23,0.09] | 0.400 | -0.03 [-0.19,0.13] | 0.688 | -0.03 [-0.20,0.15] | 0.762 | -0.04 [-0.20,0.12] | 0.595 | -0.03 [-0.19,0.12] | 0.670 |
| Emotional undereating | 0.04 [-0.12,0.20] | 0.612 | 0.03 [-0.13,0.20] | 0.710 | 0.04 [-0.12,0.21] | 0.602 | 0.06 [-0.12,0.24] | 0.483 | 0.05 [-0.12,0.22] | 0.583 | 0.04 [-0.12,0.20] | 0.623 |
| Food fussiness | 0.19 [0.02,0.37] | 0.031 | 0.18 [-0.001,0.36] | 0.052 | 0.16 [-0.02,0.34] | 0.075 | 0.24 [0.04,0.43] | 0.017 | 0.21 [0.03,0.40] | 0.021 | 0.20 [0.02,0.37] | 0.028 |
| Low physical functioning^¶^ | 1.63 [0.52,5.06] | 0.397 | 1.38 [0.44,4.33] | 0.584 | 1.63 [0.51,5.22] | 0.413 | 1.42 [0.43,4.71] | 0.567 | 1.35 [0.44,4.15] | 0.600 | 1.60 [0.52,4.98] | 0.412 |
| Low psychosocial functioning^¶^ | 2.84 [0.76,10.61] | 0.119 | 2.85 [0.75,10.84] | 0.123 | 2.83 [0.74,10.81] | 0.127 | 4.09 [0.97,17.20] | 0.054 | 2.74 [0.73,10.21] | 0.134 | 2.71 [0.72,10.18] | 0.138 |
| Physical summary score | -0.50 [-2.65,1.64] | 0.644 | 0.02 [-2.12,2.17] | 0.983 | -0.29 [-2.48,1.90] | 0.793 | -0.36 [-2.69,1.97] | 0.761 | -0.54 [-2.79,1.71] | 0.635 | -0.62 [-2.77,1.53] | 0.570 |
| Psychosocial functioning summary score | -3.04 [-5.08,-1.01] | 0.004 | -2.86 [-4.97,-0.76] | 0.008 | -2.80 [-4.88,-0.72] | 0.009 | -3.69 [-5.93,-1.45] | 0.001 | -2.83 [-4.97,-0.69] | 0.010 | -3.16 [-5.20,-1.12] | 0.003 |

Treatment effects as relative risks or mean differences and 95% confidence intervals. GA = gestational age. ^*^Pharmacological treatment using metformin or insulin or both. ^†^Trial cluster refers to hospital pairs. Socioeconomic status, determined using the NZ Deprivation Index (NZDEP) [24]. Body size determined using WHO charts [15]. ^‡^Coordination difficulty defined as a little Developmental Coordination Disorder Questionnaire score of <67 for boys and <68 for girls [16]. ^§^Total difficulties score (score ≥14) and total difficulties score relates to the Strength and Difficulties Questionnaire [17, 18]. ^||^Likely on the autism spectrum defined as ≥15 on the Social Communication Questionnaire [19]. ^¶^Low physical and psychosocial functioning defined as a score <40 on the Child Health Questionnaire PF50 [22]. NE = Not estimable.
