# Supplementary material for: Mother and child health 4.5 years after gestational diabetes mellitus managed using tight or less tight targets for glycaemic control: Post-hoc follow-up study of the TARGET trial
Source: PLoS Med. 2026 Feb 3;23(2):e1004635. doi: 10.1371/journal.pmed.1004635 (PMC12867249; doi:10.1371/journal.pmed.1004635)
Supplement: S4 Table — (DOCX) [file pmed.1004635.s004.docx]

**S4 Table:** **Post-hoc sensitivity analysis for primary maternal and child outcomes adjusted for stepped-wedge trial design.**

|  | Tight glycaemic target group | No.  n=163 mothers  n=162 children | Less tight glycaemic target group | No.  n=152 mothers n=151 children | Unadjusted treatment effect [95% CI] | Treatment effect adjusted for GA OGTT [95% CI] |
| --- | --- | --- | --- | --- | --- | --- |
| Maternal HbA1c (mmol/mol) | 40 (12.6) | 154 | 38 (8.8) | 149 | 0.05 [-2.8,2.9] | 0.69 [-2.1,3.5] |
| Maternal HbA1c (%) | 5.8 (1.6) | 154 | 5.6 (0.8) | 149 | 0.0046 [-0.25,0.27] | 0.06 [-0.19,0.32] |
| Child BMI z-score | 0.83 (1.72) | 157 | 0.75 (1.48) | 149 | 0.08 [-0.35,0.50] | 0.13 [-0.29,0.55] |

This analysis takes into account clustering within each of the pairs of hospitals within each wedge and for the time of stepping in the primary stepped-wedge trial. Data are mean (standard deviation, SD). No. = number of participants providing information for that outcome. Treatment effects are mean differences and 95% confidence intervals (CI). HbA1c = glycated haemoglobin. GA OGTT = gestational age at time of oral glucose tolerance test.
