# Supplementary material for: Mother and child health 4.5 years after gestational diabetes mellitus managed using tight or less tight targets for glycaemic control: Post-hoc follow-up study of the TARGET trial
Source: PLoS Med. 2026 Feb 3;23(2):e1004635. doi: 10.1371/journal.pmed.1004635 (PMC12867249; doi:10.1371/journal.pmed.1004635)
Supplement: S5 Table — (DOCX) [file pmed.1004635.s005.docx]

**S5 Table:** **Post-hoc sensitivity analysis: Child neurodevelopmental outcomes using a non-parametric approach (median difference) compared with a parametric (mean difference) approach.**

|  | Tight glycaemic group | No. n=162 | Less tight glycaemic group | No. n=151 | Treatment effect  Rank based median difference adjusted for GA OGTT  [95% CI] | P value | Treatment effect  Mean difference adjusted for  GA OGTT  [95% CI] | P value |
| --- | --- | --- | --- | --- | --- | --- | --- | --- |
| Little Developmental Coordination Disorder Overall Score | 72.0 [65.0,75.0] | 109 | 73.0 [70.0,75.0] | 118 | -1.00 [-2.75,0.75] | 0.263 | -2.84 [-4.99,-0.69] | 0.010 |
| -Gross motor factor subscale score | 43.0 [40.0,45.0] | 109 | 44.0 [41.0,45.0] | 118 | -1.00 [-2.47,0.47] | 0.183 | -1.44 [-2.84,-0.03] | 0.046 |
| -Fine motor factor subscale score | 29.0 [26.0,30.0] | 109 | 30.0 [29.0,30.0] | 118 | -1.00 [-1.66,-0.34] | 0.003 | -1.41 [-2.32,-0.51] | 0.002 |
| Strengths and Difficulties Questionnaire  Total difficulties score | 8.0 [5.0,10.0] | 112 | 6.0 [4.0,9.0] | 121 | 2.00 [0.78,3.22] | 0.001 | 1.75 [0.51,3.00] | 0.006 |
| Social Communication Questionnaire  Total score | 6.5 [4.0,10.0] | 108 | 5.0 [3.0,8.0] | 117 | 2.26 [0.79,3.72] | 0.003 | 1.84 [0.65,3.03] | 0.003 |
| Child Health Questionnaire PF50  Physical functioning summary score | 56.16 [53.03,58.51] | 106 | 57.42 [54.30,58.91] | 113 | -1.21 [-2.26,-0.15] | 0.025 | -0.56 [-2.70,1.57] | 0.603 |
| Child Health Questionnaire PF50  Psychosocial functioning summary score | 53.32 [48.28,57.71] | 106 | 55.90 [52.52,59.01] | 113 | -2.57 [-4.85,-0.29] | 0.027 | -3.10 [-5.13,-1.08] | 0.003 |

Results are presented as medians (Quartile 1, Quartile 3), with rank based relative effects and 95% confidence intervals (CI) for neurodevelopmental scores. GA OGTT = gestational age at time of oral glucose tolerance test.
