# Supplementary material for: Mother and child health 4.5 years after gestational diabetes mellitus managed using tight or less tight targets for glycaemic control: Post-hoc follow-up study of the TARGET trial
Source: PLoS Med. 2026 Feb 3;23(2):e1004635. doi: 10.1371/journal.pmed.1004635 (PMC12867249; doi:10.1371/journal.pmed.1004635)
Supplement: S1 Fig — (DOCX) [file pmed.1004635.s006.docx]

**S1 Figure: Box and whisker plots of primary and key child continuous neurodevelopmental outcomes.**


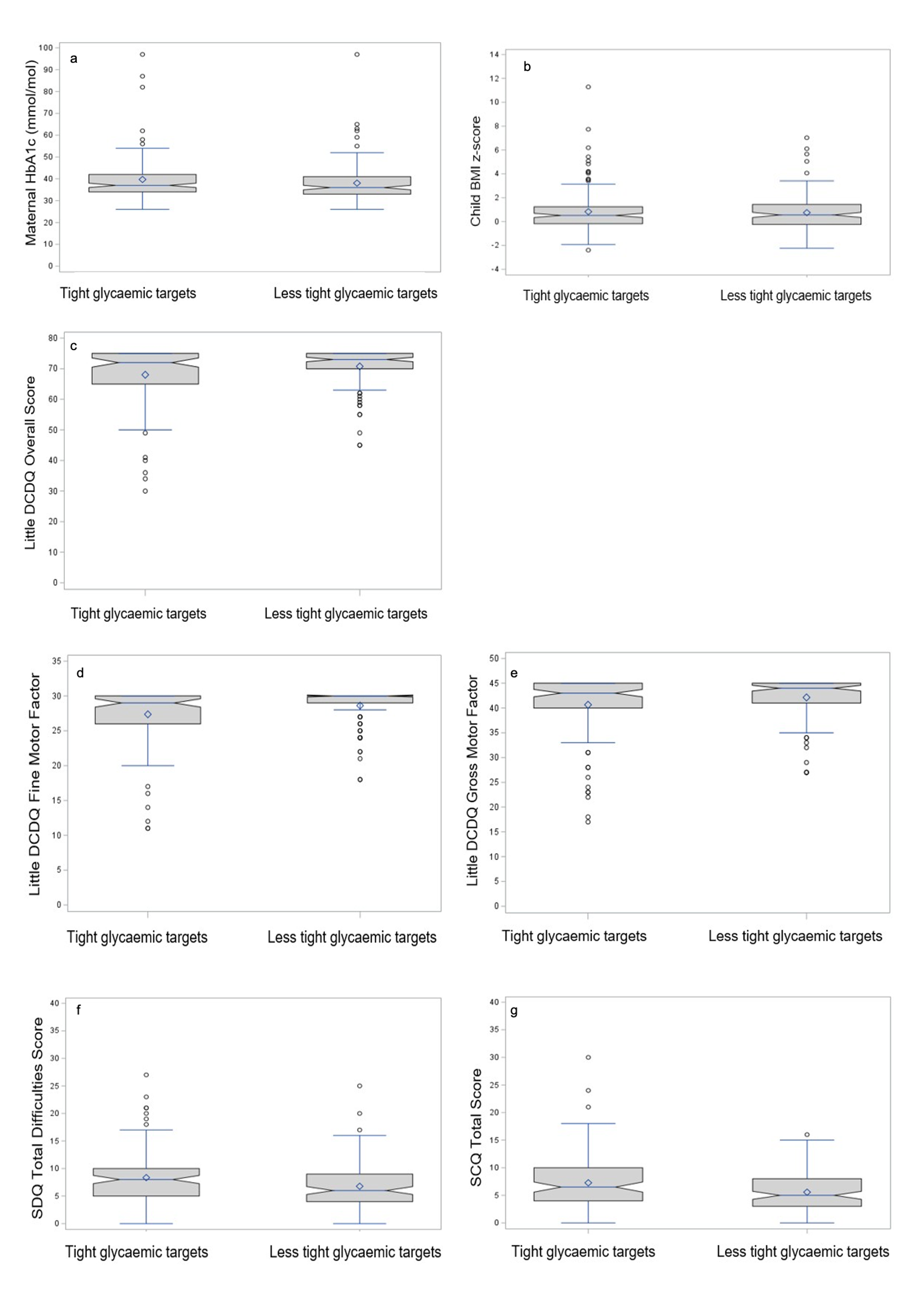


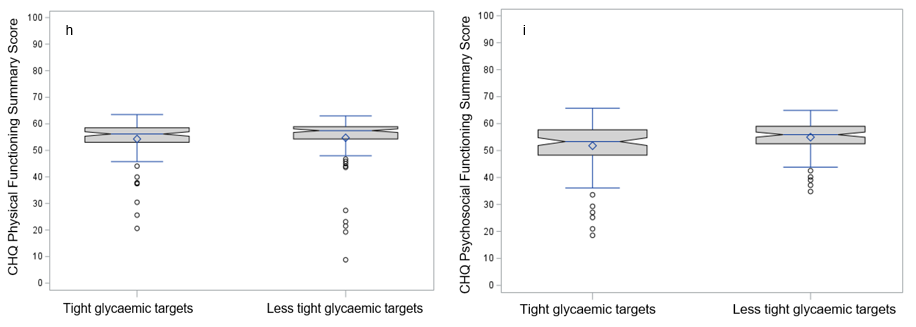


a: Maternal HbA1c (mmol/mol); b: Child BMI z-score; c: Little Developmental Coordination Disorder Questionnaire (Little DCDQ) Overall Score (maximum possible score: 75); d: Little DCDQ Fine Motor Factor (maximum possible score: 30); e: Little DCDQ Gross Motor Factor (maximum possible score: 45); f: Strengths and Difficulties Questionnaire (SDQ) Total Difficulties Score (maximum possible score: 40); g: Social Communication Questionnaire (SCQ) Total Score (maximum possible score: 39); h: Child Health Questionnaire (CHQ PF50) Physical Functioning Summary Score (maximum possible score: 100); i: CHQ Psychosocial Functioning Summary Score (maximum possible score: 100). Notched indentations on either side of the box indicate the median. Diamond the mean. The box shows the interquartile range (IQR) bottom and top at the 25th and top 75^th^ percentile respectively. Whiskers extend from the box 1.5 x IQR from the quartiles. Outliers are shown as individual points.
