# Supplementary material for: Mother and child health 4.5 years after gestational diabetes mellitus managed using tight or less tight targets for glycaemic control: Post-hoc follow-up study of the TARGET trial
Source: PLoS Med. 2026 Feb 3;23(2):e1004635. doi: 10.1371/journal.pmed.1004635 (PMC12867249; doi:10.1371/journal.pmed.1004635)
Supplement: S1 File — (DOCX) [file pmed.1004635.s007.docx]

# The Target 4.5 Year Follow-up

# Study Protocol:

Mother and Child Health after Gestational Diabetes using Tight or Less Tight Glycaemic Targets

COORDINATING CENTRE:

The Liggins Institute

The University of Auckland

85 Park Road

Grafton

Auckland 1023

Phone: +64 9 923 6011

Email: [target@auckland.ac.nz](mailto:target@auckland.ac.nz)

# **ADMINISTRATIVE INFORMATION**

**Full title:** Mother and Child Health after Gestational Diabetes using Tight or Less Tight Glycaemic Targets:

The Target 4.5 Year Follow-up Study

**Short title:** The Target 4.5 Year Follow-up Study

**Registration:** Registered with Australian New Zealand Clinical Trials Registry - ACTRN 12615000282583

**Funding:** Lottery Health Research Grant

**Host Organisation:** Liggins Institute, The University of Auckland, Auckland, New Zealand

**Investigators:**

Caroline Crowther, Liggins Institute, University of Auckland, (Principal Investigator)

Chris McKinlay, Liggins Institute, University of Auckland

Jane Alsweiler, Department of Paediatrics, University of Auckland

Greg Gamble, Liggins Institute, University of Auckland

Trecia Wouldes, Psychological Medicine, University of Auckland

Ben Thompson, University of Waterloo, Ontario, Canada

Jane Harding, Liggins Institute, University of Auckland

**Corresponding Investigator:**

Caroline Crowther

Email: c.crowther@auckland.ac.nz

Phone +64 275237795

Private Bag 92019, Auckland 1142, New Zealand

**Sites:** Liggins Institute, The University of Auckland

**Roles and responsibilities:**

Protocol development: Steering Group

Steering Group: Investigators

Management Committee: Caroline Crowther, Jane Harding, Debbie Samuel

Study Coordinator: Debbie Samuel

Data Management and Monitoring: Clinical Data Research Hub (CDRH)

Study Statistician: Greg Gamble

# **CONTENTS**

1. [INTRODUCTION](#bookmark0) 4
   1. [Background and Rationale](#bookmark1) 4
   2. [Aims and Hypotheses](#bookmark2) 5
   3. [Study Design](#bookmark3) 5
2. [METHODS](#bookmark8) 5
   1. [Participants and Study Setting](#bookmark9) 5
   2. [Assessments](#bookmark12) ……………………………………………………………………………………...6

2.2.1 Study Outcomes 6

- 1. Study Timeline………………………………………………………………………….………….6
  2. [Blinding](#bookmark20) 7
  3. [Data Collection, Management and Analysis](#bookmark21) 7
     1. [Data Collection Methods](#bookmark22) 7
     2. [Statistical Methods](#bookmark24) 7
     3. [Sample size and power](#bookmark25) 7

1. [ETHICS AND DISSEMINATION](#bookmark26) 8
   1. [Research Ethics Approval](#bookmark27) 8
   2. [Locality Approval](#bookmark28) 8
   3. [Protocol Amendments](#bookmark29) 8
   4. [Consent](#bookmark30) 8
   5. [Confidentiality](#bookmark31) 8
   6. [Participant Report](#bookmark32) 8
   7. [Withdrawal](#bookmark33) 8
   8. [Declaration of Interests](#bookmark34) 8
   9. [Access to Data](#bookmark35) 8
   10. [Dissemination Policy](#bookmark36) 9
   11. [Authorship](#bookmark37) 9
   12. [Māori Responsiveness](#bookmark38) 9
2. [STUDY MANAGEMENT](#bookmark39) 9
   1. [Steering Group](#bookmark40) 9
   2. [Management Committee](#bookmark41) 9
   3. [Standard Operating Procedures](#bookmark42) 9
   4. [Finance](#bookmark43) 9
3. [APPENDICES](#bookmark44) 10
   1. [Participant Documents](#bookmark45) 10
   2. [Case Report Forms](#bookmark46) 10
   3. [Ethical and Locality Approval](#bookmark47) 10
   4. [Protocol Amendments](#bookmark48) 10
   5. [Standard Operating Procedures](#bookmark50) 10
4. [REFERENCES](#bookmark51) 11

#

**1. INTRODUCTION**

**1.1 Background and Rationale**

***Gestational diabetes mellitus is an increasing global health problem.***

Gestational diabetes (GDM) with onset first recognised during pregnancy affects 1 in 11 pregnant women, or over 5,500 women every year in New Zealand.^1^ There is significant ethnic disparity with Māori, Pacific and Asian women at greatest risk.^1^ Internationally, as in New Zealand, the incidence of GDM has doubled in recent decades,^1,2^ the rate varying depending on the population^3,4^ and diagnostic criteria.^4-7^

***GDM contributes to an intergenerational cycle of diabetes and obesity.***

GDM is strongly associated with increased maternal and infant morbidity^8-10^ but also long-term health risks for both mother and baby, including Type 2 diabetes^11^ and the metabolic syndrome.^12,13^ With the incidence of GDM increasing,^1^ there is a perpetuating cycle of metabolic disease over generations.

***GDM is linked to later significant health problems for women and their children.***

After GDM, women have an increased risk of ischaemic heart disease,^14,15^ hypertension^15^ and Type 2 diabetes.^11,15^ One-third of women who experience GDM will develop Type 2 diabetes within 5 years, and over half within 10 years.^11^ Limited data from New Zealand suggest the risks are similar or even higher. In Christchurch, 32% of women with GDM already had impaired glucose tolerance and 14% had diabetes within 6 months after the birth.^16^ In Northland, 19% of women with GDM had diabetes by a mean of 2.4 years,^17^ and 32% by 3.9 years^18^ after the birth. Postnatal screening of all women with GDM for Type 2 diabetes is recommended by the Ministry of Health^19^ *but there is limited information on adherence to this recommendation,^20^ or the burden of disease from gestational diabetes and its long-term consequences.*

Children of women with GDM have increased risks for obesity, metabolic syndrome and diabetes,^13^ that are related to the degree of maternal hyperglycaemia.^21^ Half of infants born to mothers with diabetes develop neonatal hypoglycaemia,^22^ a risk factor for neurosensory impairment.^23^ Children exposed to maternal diabetes have an increased incidence of behavioural problems,^24^ executive dysfunction,^23^ attention deficit-hyperactivity disorder,^25^ and cognitive impairment.^26^ Motor and cognitive impairment is correlated with maternal glucose, lipid and ketone concentrations in the second and third trimesters,^27^ and may be highest in those with obesity in early childhood,^24^ suggesting that metabolic perturbations in diabetic pregnancy alter fetal brain development. *Tighter glycaemic control during pregnancy therefore may improve metabolic risk for the mothers and neurodevelopment in their children.*

***Reducing long-term health risks of GDM is a public health priority.***

Prevention of GDM has proven largely unsuccessful^28^ and rates are increasing worldwide.^1,5^ Convincing evidence from randomised trials, including our landmark ACHOIS Trial,^28^ shows that treatment for women with GDM that aims to normalise maternal glucose concentrations and reduce the fetal overgrowth related to fetal hyperglycaemia and hyperinsulinaemia reduces short-term, adverse health risks.^8,9,29^ However it remains unclear if there are long term benefits and whether the vicious intergenerational cycle of diabetes and obesity can be interrupted.^30,31^ *Effective, evidence-based interventions are urgently needed to reduce long-term cardio-metabolic and neurodevelopmental risks of GDM.*

***So what are the best glycaemic treatment targets to advise for women for GDM and can they reduce long-term health risks?***

Treatment of women with GDM with dietary advice and pharmacological support when needed is evidence based and reduces short-term morbidity.^8,9,29^ *However there are few data from randomised trials on the best glucose targets to use during pregnancy for optimal maternal glucose control,*^32^ *and minimal randomised data on the effect of tighter compared with less tight glycaemic control on the later health of women and their children.*^32,33^ International professional organisations vary in their glycaemic target recommendations for maternal glycaemic control in GDM, which all rely on consensus.^34-37^

A recent Cochrane systematic review evaluated the use of different intensities of glycaemic control for women with gestational diabetes mellitus and the effects on maternal and offspring health.^32^ Only one abstract was found which was from a randomised trial of 180 women published in 1998.^38^ In this small trial, strict glycaemic targets versus liberal glycaemic targets led to an increase in the use of insulin treatment (Relative Risk (RR) 1.85, 95% CI 1.14 to 3.03; one trial, 171 women).^32,38^ There were no significant differences between the strict and liberal glycaemic groups for macrosomia (birthweight >4000 g) (RR 1.35, 95% CI 0.31 to 5.85); birthweight (mean difference (MD) -92.00 g, 95% CI -241.97 to 57.97) or the number of small-for-gestational age infants (RR 1.12, 95% CI 0.48 to 2.63). *No data were reported for any long-term maternal or childhood outcomes.* The Cochrane systematic review concluded that to address this significant research gap “High quality trials should evaluate different blood glycaemic targets to provide evidence to guide treatment and assess both short-term a*nd long-term health outcomes for women and their babies”.*^32^

Tighter targets for glycaemic control of GDM were recommended by the Ministry of Health in the *‘Screening, diagnosis and management of gestational diabetes in New Zealand, a clinical practice guideline’* in 2014,^19^ along with a research recommendation that a randomised trial should assess the impact on the health of the mother and her child of tighter targets.^19^

**The TARGET Trial**

We have recently completed **The TARGET Trial**^39^ (Optimal Glycaemic Targets for Women with Gestational Diabetes) - a stepped-wedge, cluster-randomised trial comparing the perinatal effects of tight^19^ with less tight^40^ glycaemic targets in women with GDM. All 10 participating hospitals began using the less tight glycaemic targets (fasting blood glucose <5.5 mmol/L; 1h postprandial <8.0 mmol/L; 2h postprandial <7.0 mmol/L).^40^ Hospitals rather than women were randomised, in clusters of two, at four monthly intervals, to tighter treatment targets (fasting blood glucose ≤5.0 mmol/L; 1h postprandial ≤7.4 mmol/L; 2h postprandial ≤6.7 mmol/L).^19^ Women diagnosed with GDM at ≥22 weeks’ gestation and receiving diabetes care at the hospitals were eligible to participate.

A total of 455 women consented to participation and were recruited to the study. Differences in glycaemic control were achieved, with median fasting glucose concentrations significantly lower in women treated with tighter glycaemic targets (median 4.9 mmol/L, interquartile range (IQR) 4.6, 5.2; less tight targets median 5.0 mmol/L IQR 4.7, 5.4; P=0.05). Overall there was no difference in the primary outcome of large for gestational age (LGA) infants between treatment groups (14.7% tighter targets, 15.2% less tight targets). However, tighter glycaemic targets were associated with fewer perinatal deaths (0% versus 2.6%, P<0.05) but more serious maternal health outcomes compared with less tight glycaemic targets (5.9% versus 3.0%, P<0.02). Importantly, in the subgroup of women who were able to achieve fasting glycaemic targets ≥80% of the time, there were significantly fewer LGA infants in the tighter TARGET group (1.6% versus 12.8%, P<0.007).

*Given these findings it is essential to assess the impact of tighter glycaemic control versus less tight control on the longer-term maternal and child health and developmental outcomes, as recommended by the Ministry of Health*^19^ *and others.*^30-32^ We therefore plan to undertake The Target 4.5 Year Follow-up Study assessing mother and child health after tighter glycaemic targets for managing gestational diabetes.

**1.2 Aim and Hypotheses**

The primary aim of the Target 4.5 Year Follow-up Study is to assess whether tighter targets^19^ for glycaemic control during pregnancy for mothers with GDM compared with less tight targets^40^ reduces their later cardiometabolic risk and improves growth and development of their children 4.5 years later.

**Study hypotheses**:

Tighter targets for glycaemic control compared with less tight targets in mothers with GDM will improve:

• For the mothers – Glycaemic control; body size and composition; markers of cardiometabolic risk; dietary and activity patterns; and psychological outcomes.

• For the children – Body size and composition; neurosensory development; executive function; behaviour and health.

**Study Objectives:**

1. To determine the effects of the two different treatment targets for GDM on the later health and wellbeing of the mothers and their children.

2. To determine maternal cardiometabolic risk and growth, development and cardiometabolic risk of the children 4.5 years after GDM.

3. To determine what proportion of women adhered to the recommendation of annual type 2 diabetes (HbA1c) screening tests, what factors are associated with adherence and non-adherence to the recommendation of annual HbA1C screening, and whether adherence to HbA1c testing is associated with improved health outcomes in the mother.

**1.3 Study Design**

The Target 4.5 Year Follow-up Study is a prospective longitudinal follow-up study of a randomised trial cohort of mothers who consented to participation in the TARGET Trial and their children.

**2 METHODS**

**2.1 Participants and Study Setting**

**Eligibility**

All surviving children recruited to the TARGET Trial and their mothers who have not withdrawn from the cohort will be eligible for The TARGET 4.5 Year Follow-up Study.

**Contact Tracing**

Contact tracing will be performed using the TARGET Trial database which contains the contact details provided by mothers at the time of recruitment and at 6 months after birth. Those who have withdrawn will be marked as not eligible. A check will be made of NHI numbers to ensure we avoid contacting families whose child has died. Two months before the child reaches 4.5 years’ corrected age, families will be sent a letter, along with the Participant Information Sheet (PIS), inviting them to participate in the follow-up study and asking them to contact the study team. After one to two weeks, if we have not heard from the family, a member of research team will attempt to make contact by phone to check if the information has been received and to discuss the study. If we are unable to make contact, we will trace families via alternative contacts in the study contact database or via the primary health provider.

All consented participants will be asked to complete Mother (Health & Well-being) and Child (Health and Development) Questionnaires. Mothers will be asked to give consent for their maternal blood test results to be requested from medical records, and for their child’s health records (ie B4SC) to be accessed.

**2.2 Assessments**

***Follow-Up Protocols for the Mothers:*** Maternal questionnaires and HbA1c records from medical records for consented participants 4.5 years after the birth will allow us to determine if Type 2 diabetes has developed and whether annual HbA1c screening recommendations have been followed. Measurements of HbA1c, glucose and lipids (total cholesterol, triglycerides, LDL, HDL) from routine screening through the GP will be obtained from medical records to assess whether different intensities of glucose control influence these biological markers and are linked to later outcomes.^41^ To assess maternal psychological status and dietary and activity patterns we will use the same validated questionnaires that the mothers completed at entry into the study, at 36 weeks of pregnancy and six months after the birth on health status,^42^ anxiety,^43^ depression,^44^ diet,^45^ and physical activity,^46^ and request details of use of health services since the birth. *Weight* and *height* will be obtained from the maternal questionnaire, and where unknown, will be requested from GP records.

***Follow-Up Protocols for the Children:*** Consenting parents/caregivers will complete a questionnaire about their child’s health and development at 4.5 years’ corrected age. This age ensures assessment before the enormous but highly variable changes in physical and social environment that children experience when they start school, and allows direct comparison with findings from the B4School Check (B4SC) data requested from the Ministry of Health.

*Neurological status* (coordination, vision and hearing) are as reported by the mother or caregiver by questionnaire.

*Fine and* gross *motor function* is assessed using the Little Developmental Coordination Disorder Questionnaire (Little DCDQ).^51^

*Behavioural and emotional problems* *and functional health and well-being* are assessed using standard parentally completed questionnaires: the Strengths and Difficulties Questionnaire (SDQ)^60^ which has subscales for emotional, conduct, hyperactivity and peer problems, the Social Communication Questionnaire (SCQ)^61^ which screens for autistic spectrum behaviours, and the Child Health Questionnaire (CHQ),^62^ that assesses general health and health-related quality of life.

*Diet and physical activity and health service utilization* will be assessed by a parentally completed questionnaire that included the Child Eating Behaviour Questionniare.^63^

*Weight* and *Height* will be obtained from the Child Questionnaire, B4SC and, where unknown, will be requested from GP records.

*Tissue Management Plan;* As part of the TARGET 4.5 Year Follow-up Study, women who have not had a routine annual HbA1c testing will be encouraged to arrange this through their GP. The woman will go to her local laboratory for this test. No blood will be taken or stored as part of the TARGET 4.5 Year Follow-up Study.

**2.2.1 Study Outcomes**

**Primary outcomes**

***Independent Primary Outcomes:***

In Mothers: Glycated haemoglobin (HbA1c) at 4.5 years after the birth. (Women diagnosed with Type 2 diabetes who are currently receiving treatment for diabetes, or had a previous HbA1c ≥50mmol/mol on two separate occasions but whose HbA1C at 4.5 years after the birth is <50mmol/mol will be allocated a HbA1C of 50 mmol/mol for the analysis. Women diagnosed with prediabetes who are currently receiving treatment for prediabetes, or with a previous HbA1c 41 to 49mmol/mol but whose HbA1C at 4.5 years after the birth <41 mmol/mol will be allocated a HbA1C of 45 mmol/mol for the analysis).

In Children: Body mass index at 4.5 years of age.

#

# **Secondary outcomes**

***Secondary Outcomes for the Mothers:***

1. Diagnosis of Type 2 diabetes (currently receiving treatment for diabetes or HbA1c ≥50mmol/mol on two separate occasions) or pre-diabetes (HbA1c 41 to 49mmol/mol).^41^
2. Physical size and body composition (height, weight, body mass index (BMI)).
3. Plasma concentrations of lipids (total cholesterol, triglycerides, LDL, HDL) and fasting plasma concentrations of glucose.
4. Metabolic syndrome (defined as three or more of: hypertension; triglycerides >1.7mmol/L; HDL-cholesterol <1.29mmol/L; fasting plasma glucose >5.6mmol/L (or prediabetes or diabetes); obesity).^65,66^

5. Hypertension (defined as diagnosed by doctor and/or treated).

6. Diet quality (NZ food frequency questionnaire^45^) and physical activity (questionnaire^46^).

7. Healthcare utilisation questionnaire (postnatal diabetes screening, further pregnancies, prescriptions).

8. Health related quality of life^42^ and emotional wellbeing.^43,44^

***Secondary Outcomes for the Children:***

1. Incidence of overweight/obesity (BMI >85th centile on WHO charts).^67,68^
2. Physical size and body composition (height, weight, body mass index (BMI)).
3. Neurological status (coordination, vision, and hearing) using questionnaires completed by the mother/caregiver.
4. Motor function, using the Little DCDQ questionnaire completed by the mother/caregiver.^51^
5. Behavioural/emotional problems/functional health and well-being (Strengths and Difficulties^60^, Social Communication Questionnaire^61^, Child Health^62^ and Child Eating Behaviour Questionnaires).^63^ (SDQ: Total Difficulties Score and proportion of children with borderline or abnormal result (SDQ ≥14); Prosocial social score and proportion of children with borderline or abnormal result (score ≤5). CHQ: physical functioning and psychosocial summary scale scores and proportion more than 1 SD below normative mean for age).

**2.3 Study Timeline**

The oldest children in the Target Trial cohort turn 4.5 years in February 2020. Thus, we will aim to start recruiting as soon as ethical approval is obtained. Data collection is anticipated to be completed in 2022 after the last children in the Target Trial turn 4.5 years’ corrected age. Data analyses and publication and dissemination of findings will then be undertaken.

**2.4 Blinding**

Assessors will be blinded to the history of participants in the Target Trial. Pregnancy and postnatal data will be available to assessors after the assessment is completed only if required for clinical purposes, e.g. for clinical referrals. The intervention group allocation key will be held by the data manager until data lock and finalisation of the statistical analysis plan (SAP).

**2.5 Data Collection, Management and Analysis**

**2.5.1 Data Collection Methods**

Raw data files will be transferred to the Liggins secure servers for digital archiving. Summary scores and measures will be imported into the data management system. Where possible, parent questionnaires will be completed via electronic surveys; if hard copy questionnaires are required, data will be entered into the database at the data management centre. For fields requiring manual data entry, range and logic checks will be applied to prevent data entry errors. A Clinical Data research Hub Data Monitor will review data for completeness and logic errors. If the data monitor identifies a potential error, an electronic query will be raised referred to the relevant data entry clerk. Once data queries are resolved, data will be locked by the Data Monitor.

**2.5.2 Statistical Methods**

Statistical analysis will be performed in SAS (SAS Institute). Baseline characteristics of the cohort followed up will be compared against those lost to follow-up to inform discussion on the external validity of results. Within the follow-up cohort, intervention groups will be compared for baseline characteristics.

Primary and secondary outcomes will be compared between those exposed to less tight versus tighter glycaemic control using mixed-effects general linear modelling or logistic regression as appropriate, accounting for the stepped wedge design (random effect for hospital cluster, fixed effect for time period). Analysis will follow the intention-to-treat principle. Treatment effects will be presented as mean differences, odds ratios, or ratio of geometric means as appropriate, with 95% confidence intervals. The effect of the treatment targets on outcome will be tested. For significance tests, alpha level (two-tailed) will be set at 0.05. We will not adjust the critical alpha level for either the independent primary or secondary endpoints for multiplicity as the outcomes are in two different populations. Models will be adjusted for maternal age, socioeconomic status (NZ Deprivation Index) and gestational age at trial entry. Models without BMI as a dependent variable will be additionally adjusted for maternal BMI.

**2.5.3 Sample size and power**

The sample size is bounded by the number recruited and the follow-up rate. We will follow-up the 455 mothers who consented to the Target Trial and their children. With an estimated 85% follow-up rate, we anticipate that 387 mothers and children will participate. We will use two independent primary outcomes; one for the mothers (HbA1c) and the other for their children (BMI), each tested against a 5% significance level (90% power).

With this sample an absolute difference of 2.12 mmol/mol in mean HbA1c could be detected between the mothers in each of the groups (approximately half of one standard deviation) based on the HbA1c of women in the TARGET Trial (mean 35.0, SD 4.4 mmol/mol). A similar difference of approximately half of one standard deviation could be detected in mean BMI (absolute difference 0.96 kg/m^2^) between their children based on BMI at 4.5 years (mean 16.5, SD 2.0 kg/m^2^ ) of 140 children of mothers with GDM born at Waikato Hospital who participated in a hypoglycaemia study.^69^ Both detectable differences are likely to be clinically meaningful. PASS69 was used for the sample size calculations.^70^

**3. ETHICS AND DISSEMINATION**

**3.1 Research Ethics Approval**

Ethical approval will be sought from the Health and Disability Ethics Committee (HDEC). A progress report will be provided to HDEC annually.

**3.2 Locality Approval**

Locality approval has been sought from the Liggins Institute.

**3.3 Protocol Amendments**

All amendments to the final version of this protocol will require review and approval of the Steering Committee and will be submitted to HDEC. All amendments, including approval date, will be recorded with this protocol (Appendix 5.4).

**3.4 Consent**

Families who are interested to take part in the study and those who request further information will be sent an information pack, including the Participant Information Sheet. They will be given enough time to consider the study and to ask questions before being approached again for signed consent. Processes used to ensure confidentiality will be explained. Following this, written informed consent (hard copy or electronic) will be obtained from the mother to participate and also on behalf of the child by a caregiver. Mothers/caregivers who decline participation in the study will not be contacted again.

**3.5 Confidentiality**

The study database will be stored on a secure server at the University of Auckland and access will be controlled by unique user ID and password. Download of data will be restricted to the data management team and primary investigator. Downloaded data will be deidentified. Raw electronic data files will be stored on secure servers at the University of Auckland, accessible only to the researchers. Any hard copy records will be stored in a locked cabinet at the Liggins Institute.

Study reports will contain only summary data and individual participant data will not be reported. At the completion of the study, all electronic data will be permanently digitally archived at the Liggins Institute. Any remaining hard copy records will be stored in a locked cabinet in a secure office and will be accessible only to the study investigators. Child records will be retained for 10 years after the age of majority. All research staff will be certified in best practice for clinical trials (ICH-GCP E6 and PHRP).

The Steering Group will have access to the full dataset and oversee analysis, interpretation and reporting of results. Approval will be sought from the Steering Group prior to publication of study data. For each main publication, the corresponding data set will be electronically archived with the CDRH. Anonymised data may be shared with external researchers upon request, according to the Data Sharing Protocol of the CDRH (https://wiki.auckland.ac.nz/display/ontrack/Data+Sharing).

**3.6 Participant Report**

For those who request it on the consent form, a summary of study findings will be sent to mothers and caregivers at the time of publication of the main results.

**3.7 Withdrawal**

Mothers and caregivers will retain the right to withdraw themselves and/or their child(ren) from the study at any stage without the need to provide a reason.

**3.8 Declaration of Interests**

Investigators will declare any financial, intellectual or other potential conflicts of interest to the Steering Group. The Steering Group will decide on how any conflicts of interest are to be managed.

**3.9 Access to Data**

The Steering Group will have access to the full dataset and oversee analysis, interpretation and reporting of results. Approval will be sought from the Steering Group prior to publication of study data. For each main publication, the corresponding data set will be electronically archived with the CDRH. Anonymised data may be shared with external researchers upon request, according to the Data Sharing Protocol of the CDRH (https://wiki.auckland.ac.nz/display/ontrack/Data+Sharing).

**3.10 Dissemination Policy**

The primary mode of research dissemination will be via peer-reviewed publications, as these are most likely to impact on the research and practicing community. Most major publications are accompanied by media releases to accelerate international as well as local dissemination. Regular lectures and teaching sessions will be given to professional groups and to the public.

**3.11 Authorship**

The Council of Science Editors standards for authorship will be applied (www.councilscienceeditors.org). The Steering Group will be responsible for planning manuscripts and resolving any authorship disputes. Investigators and research staff who do not meet the criteria for authorship will be acknowledged as non-author contributors.

**3.12 Māori Responsiveness**

The TARGET 4.5 Year Follow-Up Study proposal was developed using protocols for Māori responsiveness developed by the Liggins Māori Advisory Group, University of Auckland. We have consulted with this group and will continue to work with them to develop a strategy that supports participation of Māori women to maximise the relevance for Māori health.

**4 STUDY MANAGEMENT**

**4.1 Steering Group**

The Steering Group will take overall responsibility for all aspects of the study, meeting on a bimonthly basis. Matters arising between meetings may be dealt with by email. The Principal Investigator and Study Coordinator will be responsible for maintaining a record of correspondence and minutes of meetings.

The Steering Group comprises:

Caroline Crowther, Liggins Institute, University of Auckland, New Zealand (Study Principal Investigator)

Chris McKinlay, Liggins Institute, University of Auckland

Jane Alsweiler, Department of Paediatrics, University of Auckland

Greg Gamble, Liggins Institute, University of Auckland

Trecia Wouldes, Psychological Medicine, University of Auckland

Ben Thompson, University of Waterloo, Canada

Jane Harding, Liggins Institute, University of Auckland

**4.2 Management Committee**

Ms Debbie Samuel the Study Coordinator will oversee day-to-day running of the study. She will be supported by a Management Committee that will meet regularly.

The Management Committee comprises Caroline Crowther, Jane Harding, Debbie Samuel.

**4.3 Standard Operating Procedures**

Standing operating procedures (SOPs) will be developed by the Management Committee for key study activities and processes (Appendix 5.5).

**4.4 Finance**

The Target 4.5 Year Follow-up Study is funded by a Lottery Health Research Grant.

**5 APPENDICES**

**5.1 Participant Documents**

The following participant documents are to accompany this protocol:

| Title | Version | Date |
| --- | --- | --- |
| Participant Information Form |  |  |
| Mother’s Participation Consent Form |  |  |
| Child’s Participation Consent Form |  |  |
| Maternal Questionnaire |  |  |
| Child Questionnaire |  |  |

**5.2 Case Report Forms**

The following electronic case report forms (eCRF) are to accompany this protocol:

| Title | Version | Date |
| --- | --- | --- |
| Clinical maternal assessments |  |  |
| Clinical child assessments |  |  |
|  |  |  |

**5.3 Ethical and Locality Approval**

The following letters of approval are to accompany this protocol:

| Title | Reference | Date |
| --- | --- | --- |
| HDEC national ethical approval |  |  |
| Liggins Institute locality approval |  |  |
|  |  |  |

**5.4 Protocol Amendments**

| Protocol version,  Date | Amendment(s) | Date accepted by  Steering Group | Date ethics  notified (or NA) |
| --- | --- | --- | --- |
|  |  |  |  |
|  |  |  |  |
|  |  |  |  |

**5.5 Standard Operating Procedures**

The standard operating procedures (SOP) are to accompany this protocol:

| Title | Active version | Date |
| --- | --- | --- |
|  |  |  |
|  |  |  |
|  |  |  |

**6 REFERENCES**

1. ADHB. National Women’s Hospital Annual Clinical Report. Auckland, New Zealand: http://nationalwomens health.adhb.govt.nz. 2017.

2. Feig DS, Hwee J, Shah BR, Booth GL, Bierman AS, Lipscombe LL. Trends in incidence of diabetes in pregnancy and serious perinatal outcomes. Diabetes Care 2014;37(6):1590-6.

3. Sacks D, Hadden D, Maresh M, Deerochanawong C, Dyer A, Metzger B, Lowe L, Coustan D, Hod M, Oats J, Persson B, Trimble E, HAPO Study Cooperative Research Group. Frequency of gestational diabetes mellitus at collaborating centers based on IADPSG consensus panel recommended criteria. Diabetes Care 2012; 35(3):526-28.

4. Hirst J, Tran T, Do M, Morris J Jeffrey H. Consequences of gestational diabetes in an urban hospital in Vietnam: A prospective cohort study. PLoS Med 2012; 9(7):e1001272.

5. Zhu Y, Zhang C. Prevalence of Gestational Diabetes and Risk of Progression to Type 2 Diabetes: a global perspective. Curr Diab Rep (2016) 16: 7. DOI 10.1007/s11892-015-0699-x.

6. Moses R, Morris G, Petocz P Gil F, Garg D. The impact of potential new diagnostic criteria on the prevalence of GDM in Australia. Med J Aus. 2011; 194:338-40.

7. McIntyre HD, Jensen DM, Jensen RC, Kyhl HB, Jensen TK, Glintborg D, Andersen M. Gestational Diabetes Mellitus: Does One Size Fit All? A Challenge to Uniform Worldwide Diagnostic Thresholds. Diabetes Care. 2018 Jul;41(7):1339-1342. doi: 10.2337/dc17-2393.

8. Crowther CA, Hiller JE, Moss JR, McPhee AJ, Jeffries WS, Robinson JS, and the Australian Carbohydrate Intolerance Study in Pregnant Women (ACHOIS) Trial Group. Effect of treatment of gestational diabetes mellitus on pregnancy outcomes. N Engl J Med. 2005; 352(24):2477-86.

9. Landon MB, Spong CY, Thom E, Carpenter MW, Ramin SM, Casey B, Wapner RJ, Varner MW, Rouse DJ, Thorp JM Jr, Sciscione A, Catalano P, Harper M, Saade G, Lain KY, Sorokin Y, Peaceman AM, Tolosa JE, Anderson GB, Eunice Kennedy Shriver National Institute of Child, Health Human Development Maternal-Fetal Medicine Units, Network. A multicentre, randomized trial of treatment for mild gestational diabetes. N Engl J Med. 2009; 361(14):1339-48.

10. Dodd JM, Crowther CA, Antoniou G, Baghurst P, Robinson JS. Screening for gestational diabetes: the effect of varying blood glucose definitions in the prediction of adverse maternal and infant health outcomes. Aust N Z J Obstet Gynaecol 2007;47(4):307-12.

11. Kim C, Newton KM, Knopp RH. Gestational diabetes and the incidence of Type 2 diabetes: a systematic review. Diabetes Care 2002;25(10):1862-8.

12. Boney CM, Verma A, Tucker R, Vohr BR. Metabolic syndrome in childhood: association with birth weight, maternal obesity, and gestational diabetes mellitus. Pediatrics 2005;115(3):e290-6.

13. Kawasaki M, Arata N, Miyazaki C et al. Obesity and abnormal glucose tolerance in offspring of diabetic mothers: A systematic review. PLoS ONE 2018;13(1):e0190676.

14. Sullivan SD, Umans JG, Ratner R. Gestational diabetes: implications for cardiovascular health. Curr Diab Rep 2012;12(1):43-52.

15. Daly B, Toulis K, Thomas N Gokhale K, Martin J, Webber J, Keerthy D, Jolly K, Saravanan P, Nirantharakumar K. Increased risk of ischemic heart disease, hypertension, and Type 2 diabetes in women with previous gestational diabetes mellitus. PLoS Med 2018:15(1):e1002488.

16. Hughes RC, Moore MP, Gullam JE, Mohamed K, Rowan J. An early pregnancy HbA1c >5.9% (41 mmol/mol) is optimal for detecting diabetes and identifies women at increased risk. Diabetes Care 2014;37:2953–2959.

17. McGrath N, Evans C, Holgate A. Post-partum follow-up of women with gestational diabetes mellitus from Northland, New Zealand. Diabetic Medicine 2007;24:218-9.

18. McGrath N, Baldwin A. Further post-partum follow-up of women with gestational diabetes mellitus from Northland, New Zealand. Diabetic Medicine 2012;29(3):415.

19. Ministry of Health. Screening, diagnosis and management of gestational diabetes in New Zealand. A clinical practice guideline. Ministry of Health, Wellington. 2014.

20. Hughes R, Florkowski C, Gullam J. Evaluation of the New Zealand guidelines for screening for persistent postpartum hyperglycaemia following GDM. ANZJOG 2017;1-6. DOI:10.1111/ajo.12746.

21. Ehrlich SF, Rosas LG, Ferrara A, King JC, Abrams B, Harley KG, Hedderson MM, Eskenazi B. Pregnancy glucose levels in women without diabetes or gestational diabetes and childhood cardiometabolic risk at 7 years. J Pediatr 2012;161(6):1016-21.

22. Harris DL, Weston PJ, Harding JE. Incidence of neonatal hypoglycemia in babies identified as at risk. J Pediatr 2012;161(5):787-91.

23. McKinlay CJD, Alsweiler JM, Anstice NS, Burakevych N, Chakraborty A, Chase JG, Gamble GD, Harris DL, Jacobs RJ, Jiang Y, Paudel N, San Diego RJ, Thompson B, Wouldes TA, Harding JE, for the Children With Hypoglycemia and Their Later Development (CHYLD) Study Team. Association of neonatal glycemia with neurodevelopmental outcomes at 4.5 years. JAMA Pediatrics 2017;171:972-983. doi:10.1001/jamapediatrics 2017.1579.

24. Rizzo TA, Silverman BL, Metzger BE, Cho N. Behavioral adjustment in children of diabetic mothers. Acta Paediatr 1997;86(9):969-74.

25. Nomura Y, Marks DJ, Grossman B Yoon M, Loudon H, Stone J, Halperin JM. Exposure to GDM: effects on neurocognitive development and risk of attention-deficit disorder in offspring. Arch Pediatr Adolesc Med 2012;166(4):337-43.

26. Bolanos L, Matute E, Ramirez-Duenas M, Zarabozo D. Neuropsychological impairment in school- aged children born to mothers with gestational diabetes. J Child Neurol 2015;30:1616-24.

27. Rizzo TA, Dooley S, Metzger B et al. Prenatal and perinatal influences on long-term psychomotor development in offspring of diabetic mothers. Am J Obstet Gynecol 1995;173(6):1753-8.

28. Thangaratinam S, Rogozinska E, Jolly K, Glinkowski S, Roseboom T, Tomlinson JW, Kunz R, Mol BW, Coomarasamy A, Khan KS. 2012. Effects of interventions in pregnancy on maternal weight and obstetric outcomes: meta-analysis of randomised evidence. BMJ; 344:e2088.

29. Brown J, Alwan NA, West J, Brown S, McKinlay CJ, Farrar D, & Crowther CA. Lifestyle interventions for the treatment of women with gestational diabetes. Cochrane Database of Systematic Reviews 2017, Issue 5. Art No.: CD011970. doi:10.1002/14651858.cd011970.pub2.

30. Gillman MW, Oakey H, Robinson JS, Crowther CA, Baghurst PA, Volkmer RE. Effect of treatment of gestational diabetes mellitus on obesity in the next generation. Diabetes Care 2010; 33(5), 964-968. doi:10.2337/dc09-1810.

31. Landon MB, Rice MM, Varner MW, Casey BM, Reddy UM, Wapner RJ, Rouse DJ, Biggio JR, Thorp JM, Chien E, Saade G, Peaceman AM, Blackwell SC, VanDorsten JP; Eunice Kennedy Shriver National Institute of Child Health and Human Development Maternal-Fetal Medicine Units (MFMU) Network. Mild gestational diabetes mellitus and long-term child health. 2015 Mar;38(3):445-52. doi: 10.2337/dc14-2159.

32. Martis R, Brown J, Alsweiler JM, Crawford T, Crowther CA. Different intensities of glycaemic control for women with GDM. Cochrane Database of Systematic Reviews 2016(4) Art. No.: CD011624.

33. Prutsky GJ, Domecq JP, Wang Z, Carranza Leon BG, Elraiyah T, Nabhan M, Sundaresh V, Vella A, Montori VM, Murad MH. Glucose targets in pregnant women with diabetes: a systematic review and meta-analysis. J Clin End Metab. 2013;98(11):4319-24.

34. Nankervis A MH, Moses R, Ross GP, Callaway L, Porter C, Jeffries W, Boorman C, De Vries B for the Australasian Diabetes in Pregnancy Society. Australasian Diabetes In Pregnancy Society (ADIPS) Consensus Guidelines for the Testing and Diagnosis of Gestational Diabetes Mellitus in Australia. 2013 14 February 2013.

35. American Diabetes Association. Standards of Medical Care in Diabetes—2017. Diabetes Care. 2017;40(Suppl.1): S114–S119 | DOI: 10.2337/dc17-S016

36. Scottish Intercollegiate Guidelines Network. Management of diabetes. A national clinical guideline. Edinburgh, Scotland: Scottish Intercollegiate Guidelines Network, 2010.

37. National Collaborating Centre for Women’s and Children’s Health (UK). Diabetes in Pregnancy: Management of Diabetes and its Complications from Preconception to the Postnatal Period. London: Royal College of Obstetricians and Gynaecologists, 2008.

38. Snyder J, Morin I, Melzter S, Nadeau J. Gestational diabetes and glycaemic control: a randomized clinical trial. American Journal of Obstetrics and Gynecology 1998;178(1 Pt 2):S55.

39. Crowther CA, Hiller JE, Moss JR, McPhee AJ, Jeffries WS, Robinson JS, and the Australian Carbohydrate Intolerance Study in Pregnant Women (ACHOIS) Trial Group. Effect of treatment of gestational diabetes mellitus on pregnancy outcomes. N Engl J Med. 2005; 352(24):2477-86.

40. Landon MB, Spong CY, Thom E, Carpenter MW, Ramin SM, Casey B, Wapner RJ, Varner MW, Rouse DJ, Thorp JM Jr, Sciscione A, Catalano P, Harper M, Saade G, Lain KY, Sorokin Y, Peaceman AM, Tolosa JE, Anderson GB, Eunice Kennedy Shriver National Institute of Child, Health Human Development Maternal-Fetal Medicine Units, Network. A multicentre, randomized trial of treatment for mild gestational diabetes. N Engl J Med. 2009; 361(14):1339-48.

41. New Zealand Society for the Study of Diabetes. NZSSD position statement on the diagnosis of, and screening for Type 2 diabetes. 2011. Available from www.nzssd.org.nz.

42. Ware JE, Jr, Sherbourne CD. The MOS 36-item short-form health survey (SF-36). I. Conceptual framework and item selection. Med Care. 1992 Jun;30(6):473-83.

43. Marteau TM, Bekker H. The development of a six-item short-form of the state scale of the Spielberger State-Trait Anxiety Inventory (STAI). The British journal of clinical psychology / the British Psychological Society. 1992 Sep;31 (Pt 3):301-6.

44. Cox JL, Holden JM, Sagovsky R. Detection of postnatal depression. Development of the 10-item Edinburgh Postnatal Depression Scale. Br J Psychiatry. 1987;150:782-6.

45. Sam C, Skeaff S, Skidmore P. A comprehensive FFQ developed for use in New Zealand adults: reliability and validity for nutrient intakes. Public Health Nutrition. 2012; Epub 2012 Dec 3:1-10.

46. Wendel-Vos GC, Schuit AJ, Saris WH, Kromhout D. Reproducibility and relative validity of the short questionnaire to assess health-enhancing physical activity. J Clini Epidemiol 2003;56(12):1163-9.

47. Marfell-Jones M, Norton K, Carter L, Olds T. International standards for anthropometric assessment. Lower Hutt, New Zealand: International Society for the Advancement of Kinanthropometry; 2011.

48. Sann L, Durand M, Picard J, Lasne Y, Bethenod M. Arm fat and muscle areas in infancy. Arch Dis Child. 1988;63(3):256-60.

49. Catalano PM, Thomas AJ, Avallone DA, Amini SB. Anthropometric estimation of neonatal body composition. Am J Obstet Gynecol. 1995;173(4):1176-81.

50. Gershon RC, Wagster MV, Hendrie HC, Fox NA, Cook KF, Nowinski CJ, NIH toolbox for assessment of neurological and behavioural function. Neurology 2013; 11 Suppl 3:2-6

51. Wilson, B.N., Creighton, D., Crawford, S.G., Heath, J.A., Semple, L., Tan, B., & Hansen, S. (2014). Psychometric Properties of the Canadian Little Developmental Coordination Disorder Questionnaire for Preschool Children. Physical & Occupational Therapy in Pediatrics.

52. Beery KE, Beery NA. The Beery-Buktenica development test of visual-motor integration. 6th ed. Bloomington, Minn: Pearson; 2010.

53. Yu T-Y, Jacobs R, Anstice N, Paudel N, Harding JE, Thompson B for the CHYLD Study Team. Global motion perception in two-year-old children: a method for psychophysical assessment and relationship with monocular and binocular visual function. Investigative Ophthalmology and Visual Science 30;54(13):8408-19, 2013. doi: 10.1167/iovs.13-13051.

54. Chakraborty A, Anstice NS, Jacobs RJ, Paudel, N, LaGasse LL, Lester, BM, Wouldes TA, Harding JE, Thompson B. Global motion perception is independent from contrast sensitivity for coherent motion direction discrimination and visual acuity in 4.5-year-old children. Vision Res 2015; doi: 10.1016/j.visres.2015.08.007.

55. Gerstadt CL, Hong YJ, Diamond A. The relationship between cognition and action: Performance of children 3 1/2 - 7 years old on a stroop-like day-night test. Cognition 1994; 53: 129-53.

56. Frye D, Zelazo PD, Palfai T. Theory of mind and rule-based reasoning. Cognitive Development 1995; 10:483-527.

57. Zelazo PD, Muller U, Frye D, Marcovitch S. The development of executive function in early childhood. Monographs of the Society for Research in Child Development 2003; Serial No. 264.

58. Kochanska G, Murray K, Jacques TY, Koenig AL, Vandegeest KA. Inhibitory control in young children and its role in emerging internalization. Child Dev 1996;67:490-507.

59. Reed MA, Pien DL, Rothbart MK. Inhibitory self-control in preschool children. Merill-Palmer Quarterly 1984;30(2):131-47.

60. Goodman R. The Strengths and Difficulties Questionnaire. J Child Psych Psychiat 1997; 38: 581-6.

61. Rutter M, Bailey A, Lord C. Social Communication Questionnaire. Torrance, CA: Western Psychological Services; 2003.

62. Landgraf JM, Abetz L, Ware JE. Child Health Questionnaire (CHQ): A user's manual. 1996. Boston, MA: The Health Institute, New England Medical Center.

63. Wardle J, Guthrie CA, Sanderson S, Rapoport L. Development of the Children's Eating Behaviour Questionnaire. J Child Psychol Psyc. 2001;42:963-970. 10.1111/1469-7610.00792.

64. Rowan JA, Rush EC, Obolonkin V, Battin M, Wouldes T, Hague WM. Metformin in gestational diabetes: the offspring follow-up (MiG TOFU): body composition at 2 years of age. Diabetes Care. 2011; 34(10):2279-84.

65. Alberti KG, Eckel RH, Grundy SM, Zimmet PZ, Cleeman JI, Donato KA, Fruchart JC, James WP, Loria CM, Smith SC Jr; International Diabetes Federation Task Force on Epidemiology and Prevention; National Heart, Lung, and Blood Institute; American Heart Association; World Heart Federation; International Atherosclerosis Society; International Association for the Study of Obesity. Harmonizing the metabolic syndrome. Circulation 2009;120(16):1640–5.

66. Health Navigator New Zealand 2022. https://healthnavigator.org.nz.

67. Barlow SE, Dietz WH. Obesity evaluation and treatment: expert committee recommendations. Pediatrics. 1998; 102 (3): e29.

68. NZ WHO Growth Charts. https://www.health.govt.nz/system/files/documents/pages/factsheet-2-growth-charts-well-child.pdf.

69. Harris DL, Weston PJ, Signal M, Chase JG, Harding JE. Dextrose gel for neonatal hypoglycaemia (the Sugar Babies Study): a randomised, double-blind, placebo-controlled trial. Lancet 2013;382:2077-83.

70. PASS (Power Analysis and Sample Size Software (2018). NCSS, LLC. Kaysville, Utah, USA, ncss.com/software/pass).
