# Supplementary material for: Mother and child health 4.5 years after gestational diabetes mellitus managed using tight or less tight targets for glycaemic control: Post-hoc follow-up study of the TARGET trial
Source: PLoS Med. 2026 Feb 3;23(2):e1004635. doi: 10.1371/journal.pmed.1004635 (PMC12867249; doi:10.1371/journal.pmed.1004635)
Supplement: S2 File — (DOCX) [file pmed.1004635.s008.docx]

Optimal glycaemic targets for gestational diabetes: TARGET 4.5 Year Follow-up Study

| Statistical Analysis Plan  Health data Primary | |  |
| --- | --- | --- |
| Protocol Title | The TARGET 4.5 Year Follow-up | |
| Protocol Date: | 9^th^ August 2022, version 1.2 | |
| SAP Authors: | Lisa Douglas, Greg Gamble | |
| SAP Version: | 1.1 | |
| SAP Date: | 14 June 2023, v 1.0  25 October 2024, v 1.1 | |

TABLE OF CONTENTS

[1. Preface 6](#_Toc137560917)

[2. Purpose of SAP 6](#_Toc137560918)

[3. Study aims, hypotheses and endpoints 6](#_Toc137560919)

[3.1 Study aims 6](#_Toc137560920)

[3.1.1 Primary study aim 6](#_Toc137560921)

[3.1.2 Secondary study aims 7](#_Toc137560922)

[3.2 Study hypotheses 7](#_Toc137560923)

[3.2.1 Primary hypotheses 7](#_Toc137560924)

[3.2.2 Secondary hypotheses 7](#_Toc137560925)

[3.3 Study endpoints 7](#_Toc137560926)

[3.3.1 Primary endpoints: 7](#_Toc137560927)

[3.3.2 Secondary endpoints 8](#_Toc137560928)

[4. Study methods 12](#_Toc137560929)

[4.1 Overall study design and plan 12](#_Toc137560930)

[4.2 Selection of study population 12](#_Toc137560931)

[5. Sequence of planned analysis 12](#_Toc137560932)

[5.1 Interim analyses 12](#_Toc137560933)

[5.2 Final analyses and reporting 12](#_Toc137560934)

[6. Power determination 13](#_Toc137560935)

[7. General issues for statistical analysis 14](#_Toc137560936)

[7.1 Analysis software 14](#_Toc137560937)

[7.2 Analysis approach 14](#_Toc137560938)

[7.3 Methods for withdrawals, missing data, and outliers 14](#_Toc137560939)

[7.4 Protocol violations and deviations 14](#_Toc137560940)

[7.5 Data transformations 14](#_Toc137560941)

[7.6 Potential confounders 14](#_Toc137560942)

[7.7 Planned treatment by covariate interactions 15](#_Toc137560943)

[7.8 Multiple comparisons and multiplicity 15](#_Toc137560944)

[8. DESCRIPTIVE analysis 16](#_Toc137560945)

[8.1 Flow chart of participants 16](#_Toc137560946)

[8.2 Baseline characteristics 16](#_Toc137560947)

[8.3 Missing data 16](#_Toc137560948)

[9. STATISTICAL analysis 16](#_Toc137560949)

[10. SHELL TABLES 18](#_Toc137560950)

[10.1 Figure 1: Flow chart of participants in the TARGET 4.5 Year Follow-up Study. 18](#_Toc137560951)

[10.2 Table 1: Baseline TARGET Trial entry characteristics of women who were eligible for follow-up at 4.5 years and included or not included. 19](#_Toc137560952)

[10.3 Table 2: Baseline characteristics of mothers and children included in the 4.5 Year Follow-up. 21](#_Toc137560953)

[10.4 Table 3: Primary and secondary outcomes among the mothers at 4.5 Year Follow-up. 24](#_Toc137560954)

[10.5 Table 4: Primary and secondary outcomes among the children at 4.5 Year Follow-up. 30](#_Toc137560955)

[11. REFERENCES 34](#_Toc137560956)

ABBREVIATIONS

| **ABBREVIATION** | **DEFINITION** |
| --- | --- |
| B4School Check | Before School Check |
| BMI | Body Mass Index |
| CEBQ | Child Eating Behaviour Questionnaire |
| CHQ-PF28 | Child Health Questionnaire |
| EDD | Estimated Delivery Date |
| FPG | Fasting Plasma Glucose |
| GA | Gestational Age |
| GDM | Gestational Diabetes Mellitus |
| GLMM | Generalised Linear Mixed Model |
| GMFCS | Gross Motor Function Classification System |
| GP | General Practitioner |
| HbA1c | Glycated Haemoglobin |
| HDL | High Density Lipoprotein |
| ICC | Intra-cluster correlation |
| ITT | Intention-To-Treat |
| LDL | Low Density Lipoprotein |
| LGA | Large for Gestational Age |
| Little DCDQ | Little Developmental Coordination Disorder Questionnaire |
| NZ | New Zealand |
| NICU | Neonatal Intensive Care Unit |
| OGTT | Oral Glucose Tolerance Test |
| SAP | Statistical Analysis Plan |
| SCQ | Social Communication Questionnaire |
| SD | Standard Deviation |
| SDQ  SGA | Strengths and Difficulties Questionnaire  Small for Gestational Age |
| TARGET Trial | Optimal glycaemic targets for gestational diabetes, tight or less tight glycaemic targets for women with gestational diabetes mellitus for reducing maternal and perinatal morbidity |
| WHO | World Health Organization |

#

# Preface

This Statistical Analysis Plan (SAP) describes the planned analysis and reporting for the TARGET 4.5 Year Follow-up Study.

The following documents were reviewed in preparation of this SAP:

- The TARGET Protocol version 5.0 (17Sep2014) (1)
- The TARGET Trial SAP version 3.0 (17Apr2018)
- The TARGET Trial paper (published 8Sep2022) (2)
- The TARGET 4.5 Year Follow-up Study Protocol version 1.2 (9Aug2022)
- TARGET 4.5 Year Follow-up data collection forms.

The reader of this SAP is encouraged to also read the clinical protocols for details on the conduct of this study, and the operational aspects of clinical assessments and timing for a participant in this study.

The TARGET 4.5 Year Follow-up Study Protocol lists the Steering Group (section 4.1), and study management group (section 4.2).

# Purpose of SAP

The purpose of this SAP is to outline the planned analyses to be completed to support the completion of papers for the TARGET 4.5 Year Follow-up Study that will include for the mother: glycaemic control, body size and cardiometabolic risk; dietary and activity patterns; psychological outcomes; adherence with postpartum diabetes screening recommendations; and for the child: body size, neurosensory development; cardiometabolic risk, behaviour and health. These may be reported in more than one manuscript.

The SAP will not cover any side studies, nor cost analyses nor any in-depth studies on dietary and activity patterns, psychological outcomes and adherence with postpartum diabetes screening recommendations.

# Study aims, hypotheses and endpoints

The background and rationale for 4.5 year follow-up of mothers and babies from the Target Trial are provided in the study protocol (section 1.1).

## 3.1 Study aims

### 3.1.1 Primary study aim

The primary aim of the Target 4.5 Year Follow-up Study is to assess whether tighter targets for glycaemic control during pregnancy for mothers with gestational diabetes mellitus (GDM) compared with less tight targets reduces their later cardiometabolic risk and improves growth and development of their children at 4.5 years. Tighter targets were fasting plasma glucose ≤5mmol/L (≤90mg/dL), 1 hour ≤7.4mmol/L (≤133mg/dL), 2 hour ≤6.7mmol/L (≤121mg/dL) and less tight targets were fasting plasma glucose <5.5mmol/L (<99mg/dL), 1 hour <8mmol/L (<144mg/dL) and 2 hour <7mmol/L (<126mg/dL).

### 3.1.2 Secondary study aims

1. To assess whether tighter targets for glycaemic control during pregnancy for mothers with GDM compared with less tight targets affect maternal dietary and activity patterns and psychological outcomes.

2. To assess whether tighter targets for glycaemic control during pregnancy for mothers with GDM compared with less tight targets affect adherence to the recommendations for postnatal HbA1C screening.

## 3.2 Study hypotheses

### 3.2.1 Primary hypotheses

Tighter targets for glycaemic control compared with less tight targets in mothers with GDM will improve:

• For the mothers – Glycaemic control; body size and markers of cardiometabolic risk;

• For the children – Body size (height, weight and body mass index), neurological status, motor function, behavioural and emotional health, and functional health and wellbeing.

### 3.2.2 Secondary hypotheses

Tighter targets for glycaemic control compared with less tight targets in mothers with GDM will improve maternal dietary and activity patterns and psychological health.

Tighter targets in pregnancy for glycaemic control compared with less tight targets in mothers with GDM will not affect the proportion of women who adhere to the recommendation of annual type 2 diabetes (HbA1c) screening tests.

## 3.3 Study endpoints

### 3.3.1 Primary endpoints:

***Independent Primary Outcomes:***

*I****n Mothers: HbA1c at 4.5 years after the birth.***

Women diagnosed with Type 2 diabetes who are currently receiving treatment for diabetes, or had a previous HbA1c ≥50mmol/mol on two separate occasions but whose HbA1c at 4.5 years after the birth is <50mmol/mol will be allocated a HbA1c of 50 mmol/mol for the analysis. Women diagnosed with prediabetes who are currently receiving treatment for prediabetes, or with a previous HbA1c of 41 to 49mmol/mol but whose HbA1c at 4.5 years after the birth <41 mmol/mol will be allocated a HbA1c of 45 mmol/mol for the analysis. A medical adjudication panel blinded to original study randomisation groups will inspect the available HbA1c results and, based on proximity to 4.5 years after birth, and self-reported diabetes or prediabetes apply the above criteria and determine the appropriate value to include in the analysis.

***In Children: Body mass index (BMI)z-score at 4.5 years corrected age (continuous outcome)****).*

BMI z-score for corrected age will be calculated using WHO charts (3). Children where anthropometric measurements were made only at <3 or >6 years of age will be excluded from this analysis. Values of weight and height to include in the analysis will be based on proximity to 4.5 years after birth and prioritised as below. This outcome will be derived from the Before School check (B4SC) (4) or reported weight and height (Child Questionnaire: A1 Child’s most recent weight (in last 6 months), A2 Child’s height, A3 Date of measurements), or weight and height obtained from GP records. A medical adjudication panel blinded to original study randomisation groups will inspect available body size data for individual children and determine the appropriate value to include in the analysis, prioritising BMI results according to this hierarchy:

1. B4SC between 4 to 5 years.

2. GP/ Other sources/ or in person assessment between 4 to 5 years.

3. Parent report between 4 to 5 years.

4. B4SC between <4 or > 5 years.

6. GP/ Other sources/ or in person assessment <4 years or > 5 years.

7. Parent report <4 years or > 5 years.

Where a participant has two or more estimates of BMI that differ by >3.5kg/m^2^ (5) the individual results will be checked against source records and, if necessary, flagged for the adjudication panel review.

### 3.3.2 Secondary endpoints

***Secondary outcomes for the mothers:***

*1. Diagnosis of Type 2 diabetes* (currently receiving treatment for diabetes or HbA1c ≥50mmol/mol on two separate occasions) or self-reported diabetes. (Q13 maternal questionnaire) or *pre-diabetes* (HbA1c 41 to 49mmol/mol) (6) by laboratory testing or self-reported pre-diabetes (Q14 maternal questionnaire).

*2. Diagnosis of Type 2 diabetes* (currently receiving treatment for diabetes or HbA1c ≥50mmol/mol on two separate occasions) or self-reported diabetes. (Q13 maternal questionnaire).

3. *Diagnosis of pre-diabetes* (HbA1c 41 to 49mmol/mol)(6) by laboratory testing or self-reported pre-diabetes (Q14 maternal questionnaire).

4. *Body size* (height, weight, and BMI). The outcomes will use height and weight self-reported or from GP records. If height not available from these sources, height from the TARGET Trial will be used.

*5. Plasma concentrations of lipids* (total cholesterol, triglycerides, low density lipoprotein (LDL), high density lipoprotein (HDL), total cholesterol/HDL ratio (cholesterol ratio) (maternal data from medical records Q24-40).

6. *Hypertension* (self-reported high blood pressure diagnosed by a doctor and/or treated) (maternal questionnaire Q18=Yes and/or Q19=Yes).

7. *Metabolic syndrome* (defined as three or more of: hypertension; triglycerides >1.7mmol/L; HDL-cholesterol <1.29mmol/L; FPG >5.6mmol/L; (laboratory results from maternal information medical records form) prediabetes or diabetes; obesity (BMI >30kg/m^2^ using height from TARGET Trial and self-reported weight at follow-up) (7, 8).

8. *Self-reported healthcare utilisation after birth* including further pregnancies (maternal questionnaire Q4=Yes), and if so number of further pregnancies (Q5), further pregnancies complicated by gestational diabetes (Q6=Yes) or high blood pressure or pre-eclampsia (Q7=Yes) or pre-term birth (Q8=Yes); prescriptions for the treatment of diabetes (Q17.2=Yes and/or Q17.3=Yes), diagnosis of diabetes (Q13=Yes), hypertension (Q19=Yes), or dyslipidemia (Q21=Yes). Cardiovascular or cerebrovascular events Q22 to Q27=yes and other major illnesses (Q2=Yes) will be described.

9. *Maternal dietary macronutrients* will be assessed using the New Zealand food frequency questionnaire (9). The dietary food frequency questionnaire consisted of 57 food items to assess maternal food intake over the past month and analysed by selected macronutrients: Total energy, total carbohydrate, total fat and protein.

10. *Maternal physical activity patterns* will be assessed using the SQUASH physical activity questionnaire (10) using, as a continuous outcome the following activities: communication, leisure time, household, school/work and total activities scores; and metabolic equivalent of task (METs) reported categorically with light intensity <4 METs, moderate intensity 4 to 6.5 METs, and vigorous intensity >6.5 METs.

11. *Maternal psychological health;* Health related quality of life assessed using the 36-item short-form (SF-36 version 2) (11) and emotional wellbeing with the Edinburgh Postnatal Depression Scale (EPDS) (12) and the Spielberger State-Trait Anxiety Inventory (STAI) (13). To assess the adequacy of response for the psychometric instruments the proportion of women completing each instrument and the completeness of each record will be reported. Reliability statements from each of the psychometric instruments will be compiled.

The SF-36 has questions on 8 health concepts, with a single question on perceived change in health. An overall physical component and an overall mental component will be calculated (11). Continuous mother level outcome for each of the following subscales of the SF-36: physical functioning, physical role, bodily pain, general health, vitality, social functioning, emotional role, mental health, overall physical component and overall mental component (SF-36 questionnaire) will be calculated. The scores will be computed using SF-36 scoring processes for the data collected.

The EPDS is a validated 10 question tool used to identify postnatal depression (12). Questions are scored 0-3, with the highest mark given to the answer indicating the most distress, with a maximum score of 30. Continuous mother level outcome and binary mother level outcome for postnatal depression is defined by an EPDS score >12.

The STAI is a measure of anxiety, with the six item short form covering the three highest anxiety-present and anxiety-absent items from the full 20-item STAI (13). The short form had similar scores and acceptable reliability. Questions are scored from 1 (not at all) to 4 (very much). Continuous mother level outcome defined using Anxiety scoring process and binary mother level outcome defined by a short form of the STAI score ≥15 as being abnormal (14).

12. *Adherence with postpartum diabetes screening recommendations.*

The proportion of women who had HbA1c measurements at 3 months post-partum (6 weeks to 6 months) AND who subsequently had annual HbA1c measurements (+/- 6 months) will be documented, up until the time at which they were diagnosed with Type 2 Diabetes. Comparison also will be made of the proportion of recommended HbA1c tests that were completed prior to any diagnosis of diabetes. Categorical outcomes will be reported of the following: any HbA1c measurement, one HbA1c measurement, two HbA1c measurements, three HbA1c measurements, four HbA1c measurements or five HbA1c measurements, completed at any time during the 4.5 year follow-up. The diagnosis of Type 2 Diabetes requires two abnormal tests (HbA1c ≥50mmol/mol) in an asymptomatic adult which can be done with no delay between tests (15).

***Secondary outcomes for the children:***

1. *Incidence of children overweight/obese* (BMI z score > + 2 using WHO charts and corrected age (3, 16); incidence of overweight (BMI z score > +2 and < +3) (16); and incidence of obesity (BMI z score > +3) (16). BMI obtained from the B4 School Check, child questionnaire, or if required, from GP records, as outlined for primary outcome section 3.3.1).

2. *Other body size measures will be assessed* by height; height z-score; short stature (height z-score < -2 (16)); weight; and weight z-score categories (< -2, -2 to +2, > +2 and ≤ +3, and >+3). Height and weight z scores will be based on corrected age.

3. N*eurological status* (cerebral palsy, blindness, deafness, developmental delay, coordination, other visual problems and other hearing problems) using questionnaires completed by the mother/caregiver. Cerebral palsy Q7=yes; blindness Q8 2.3 or 2.4=yes; deafness Q10 2.2 or 2.3=yes; developmental delay Q11=yes; coordination Q3-6 (difficulty walking Q3=yes, difficulty sitting Q4=yes, difficulty using hands Q5 = yes, difficulty with head control Q6 =yes); or other visual problems Q8 2.1 or 2.2=yes and/or Q9=yes; and other hearing problems Q10 2.1=yes; attending special care programme Q12=yes; receiving supportive care Q13=yes, and type of care Q13.2.1-13.2.5. A composite for neurosensory disability will comprise any of cerebral palsy, blindness, deafness or developmental delay.

4. *Fine and gross motor function* will be assessed using the Little Developmental Coordination Disorder Questionnaire (DCDQ) completed by the mother/caregiver (17). Continuous and dichotomous scoring, using a total score for boys and a total score for girls, and cutoffs < 67 for boys and < 68 for girls. Gross motor factor subscale score (sum of items 1,2,3,4,5,12,13,14,15) and fine motor factor subscale score (sum of items 6,7,8,9,10,11) (18).

5. *Behavioural and emotional problems* will be assessed by the Strengths and Difficulties Questionnaire (SDQ) (19), Social Communication Questionnaire (SCQ) (20) and Child Eating Behaviour Questionnaire (CEBQ) (21). To assess the adequacy of response for the psychometric instruments the proportion of women completing each instrument and the completeness of each record will be reported. Reliability statements from each of the psychometric instruments will be compiled.

The SDQ has subscales for emotional symptoms, conduct problems, hyperactivity, peer relationship problems and pro social behaviour (19). Questions are scored 0 to 2 according to the scoring key. A total difficulties score will be generated by summing the scores (except the prosocial score), ranging from 0 to 40. There are five additional short impact supplement questions. Dichotomous scoring will be prioritised with a score of ≥14 on the total difficulties score considered borderline or abnormal (22). Total and subscale scores will also be reported. (SDQ obtained from the questionnaire or, where not available, from the B4SC if available).

The SCQ is used to screen for autistic spectrum behaviours (20). Each question requires a “yes” or “no” response with one point scored for each abnormal behaviour. Only verbal children with a “yes” response to the first question answer the six items relating to abnormal language and can score a total of 0–39 points. Non-verbal children are not assigned the six items in relation to language and can score a total of 0–33 points. Dichotomous scoring will be prioritised with a cut-off of ≥15 indicating that the child is likely to be on the autism spectrum and requires further evaluation, with this cut-off giving a sensitivity and specificity of 0.89 (23). We have chosen a score of ≥15 as this higher cut-off has a higher specificity for a diagnosis of autism spectrum disorder (24, 25). Total scores will also be reported.

The CEBQ is presented as a mean (standard deviation) for each eating style assessed on eight domains: food responsiveness (4 items), enjoyment of food (4 items), emotional overeating (4 items), desire to drink (3 items), satiety responsiveness (5 items), slowness in eating (4 items), and emotional undereating (4 items), and fussiness (7 items) (26). Scoring is using a 5-point scale: 1 = never, 2 = rarely, 3 = sometimes, 4 = often, 5 = always. Only those domains with at least 80% of the questions completed will be included.

6. *Functional health and wellbeing will be* assessed using the Child Health Questionnaire (CHQ) (27). The CHQ assesses general health and health related quality of life CHQ: physical functioning and psychosocial summary scale scores and proportion more than 1 SD below normative mean for age (mean=50 SD=10) will be reported.

# 4. Study methods

## 4.1 Overall study design and plan

The Target 4.5 Year Follow-up Study is a prospective longitudinal follow-up study of a randomised trial cohort of mothers who consented to participation in the TARGET Trial and their children. The TARGET Trial (2) is a multi-centre, stepped-wedge, cluster, randomised trial (28-33).

## 4.2 Selection of study population

Eligibility Criteria: All mothers with a singleton pregnancy who gave consent to the TARGET Trial and their children, who had not withdrawn from further follow up, where birth data were available, mother and child were known to be alive and where the child was not in care outside the family were considered eligible for The TARGET 4.5 Year Follow-up Study.

# 5. Sequence of planned analysis

## 5.1 Interim analyses

No interim analysis was planned or conducted.

## 5.2 Final analyses and reporting

Data will be analysed by a statistician and doctoral student. Once tracking is completed and consents and data are obtained, blinded review of the data will be performed to inform collection of HbA1c, plasma glucose and lipid results from general practices. B4School check data will be requested from the Ministry of Health. The collection of child and maternal height and weight results from the GP when these data are missing from the Mother and Child questionnaires and requests for B4 School Check data will continue while mother and child questionnaires are returned and processed. Blinded review of the data without knowledge of treatment allocation will be conducted, and final changes will be made to this SAP prior to data lock. Following the approval of the final version of this SAP, analysis of the study outcomes will be performed blinded to the treatment allocation before treatment allocation unlock and completion of the final analysis. Blinded review of HbA1c results will be made by a data adjudication panel to identify the most appropriate HbA1c result to include in the analysis, and for the diagnosis of Type 2 diabetes, prediabetes, and metabolic syndrome. An adjudication panel will also review the body size measures (height and weight) for children to identify the most appropriate measure to include in the analyses. Key statistics and study results will be made available to the study investigators. Any post-hoc, exploratory analyses which were not identified in this SAP but are completed to support the planned analyses will be clearly identified. Any deviations from the planned analyses detailed in this SAP will be clearly documented with reasons in a post-analysis version of the SAP.

# 6. Power determination

It was originally expected that 85% of the original study cohort (387/455 consenting mothers) would consent to follow-up. This power determination has been recalculated at the end of follow-up on the basis of 315 mothers (76 % of the eligible trial cohort) and 313 children for whom consent to participate in the TARGET 4.5 Year follow-up study was obtained. Inspection of the number of participants within each of the original TARGET trial two hospital clusters showed some clusters had too few participants in either or both of the intervention arms to proceed with a step wedge analysis of data at 4.5 years follow-up. Without consideration of the stepped-wedge design, assuming an equal variance and a mean HbA1c in the less tight target group of 35 mmol/mol (SD=4.4) based on the HbA1c of women in the TARGET Trial, a difference of at least 1.6 mmol/mol (36% of one standard deviation) between the less tight and tight target groups could be detected. In their children based on BMI at 4.5 years (mean 16.5 kg/m^2^, SD 2.0) of 140 children of mothers with GDM born at Waikato Hospital who participated in a hypoglycaemia study (34) differences of 0.7 kg/m^2^ (36% of one standard deviation) could be detected. These effect sizes are estimated for 90% power at the 5% significance level using PASS 16 (PASS 16 Power Analysis and Sample Size Software (2018)). NCSS, LLC. Kaysville, Utah, USA (35).

# 7. General issues for statistical analysis

## 7.1 Analysis software

All analysis will be performed using SAS software version 9.4 or later (SAS Institute Inc., Cary, NC, USA).

## 7.2 Analysis approach

The planned analyses will be carried out using the intention-to-treat approach in which participants will be analysed according to the treatment targets group to which the hospital where they received care was randomised. Prior to unblinded analysis the proportion of participants in each step-wedge cluster was examined and there were too few participants to permit analysis according to the step-wedge design. The primary analysis approach will therefore be an independent groups analysis without adjustment for clustering and time of randomization on the basis that the models are unlikely to converge with sparse intra-cluster data.

## 7.3 Methods for withdrawals, missing data, and outliers

We will include data for all participants who consented to participate in this follow-up. For children who were <3 or >6 years corrected age at time of their only weight and height measurements those measurements will be considered missing. Overall pattern of missingness, including amount, distribution across study groups and over different outcomes, and covariates to be adjusted for will be examined. No imputation will be performed because the assumption of missing-at-random is unlikely to be met.

## 7.4 Protocol violations and deviations

No subjects will be excluded from the intention-to-treat analyses due to protocol violations or deviations.

## 7.5 Data transformations

No data transformations are planned. Data transformation may be investigated if assumptions about the distribution of the outcomes are invalid. Data transformations are not planned to correct for departures from normality, since the sample size is sufficient for the central limit theorem to apply (36).

## 7.6 Potential confounders

Both unadjusted and adjusted analyses will be carried out for each outcome. Conclusion about the effect of treatment will be drawn from the adjusted results; meanwhile the unadjusted analysis will be used to potentially confirm the results of the adjusted analyses.

Analyses will make adjustment for gestational age (GA) at time of oral glucose tolerance test (OGTT) as the pre-defined covariate. Secondary exploratory analyses will consider important baseline predictors identified during the analysis which show evidence of substantial imbalance between the study groups and are related to the outcome of interest.

| **Pre-defined potential confounding variables** | **Details** |
| --- | --- |
| Gestational age at time of OGTT | Continuous variable calculated from estimated delivery date (EDD) and date of OGTT done. If a linear relationship with GA at time of OGTT is not appropriate, either a quadratic term will be added (if a quadratic relationship appears reasonable), or a continuous variable will be dichotomised (< 28 weeks vs. ≥ 28 weeks). |

If convergence is an issue, potential confounders may need to be collapsed into fewer categories or excluded from the adjusted analysis for particular outcome(s). Any deviation from the planned adjustment for potential confounders will be clearly identified.

## 7.7 Planned treatment by covariate interactions

Evidence of effect modification will be inspected for the primary and secondary outcomes. The analysis will be carried out to test for evidence of effect modification. For mothers the following will be investigated: HbA1c; diagnosis of diabetes or diagnosis of prediabetes; and metabolic syndrome at 4.5 years after birth. For children BMI-Z score; incidence of overweight or obesity at 4.5 years of age. Each will be explored by including interaction terms in the model for the variable and the planned covariates listed below. For categorical variables, separate estimates of treatment effect will be obtained within each category and categories pooled when the numbers within each category are found to be small. For continuous variables, interactions will be presented graphically, independent of whether the interaction is statistically significant or not, since these carefully chosen limited comparisons are of interest *a priori*. Any unplanned treatment by covariate interactions must be considered exploratory and will be clearly identified. The classification of planned covariates is as follows: gestational age at birth (<37 completed weeks’ (pre-term) and >37 completed weeks’ (term)), maternal ethnicity (Māori, Pacifica, Asian, Other or NZ European,) management of diabetes (diet alone vs pharmacological management with any of metformin, insulin or both metformin and insulin).

## 7.8 Multiple comparisons and multiplicity

The primary outcomes for both mother and child will be assessed at P<0.05 with no adjustment for multiplicity. Multiple hypothesis tests will need to be performed to assess the effectiveness of the intervention on multiple secondary outcomes, unadjusted and adjusted analyses, and planned treatment by covariate interactions.

No adjustment to the critical significance level will be used for the secondary outcomes or any planned treatment by covariate interactions. As a result, any nominally significant results will be interpreted and reported with caution and considered in the context of consistency and biological plausibility.

# 8. DESCRIPTIVE analysis

## 8.1 Flow chart of participants

This will show the flow of participants through the study and will include the number of mothers with a singleton pregnancy who gave consent to participate in the original TARGET trial and their children. These women and their children were considered eligible for the TARGET 4.5 year follow up study if their birth data were available, and, prior to hospital discharge after the birth, they had not withdrawn for further follow up, mother or child were known to be alive, and the child was not in care outside of the family. Not eligible for follow-up were women or children who had died since hospital discharge after the birth, or where the child was now in care outside the family. The number of women and/or children eligible for follow-up, where consent was declined, and women and/or children unable to be contacted will be shown. The number of women and children in the tighter and less tight glycaemic target groups eligible for and included in the Target 4.5 Year Follow-up Study will be shown (Figure 1).

## 8.2 Baseline characteristics

Baseline characteristics of all eligible women will be compared descriptively between the study groups, less tight and tighter glycaemic target periods (see shell tables) to assess comparability of groups. Means and standard deviations, or medians and interquartile ranges will be reported for continuous variables where appropriate. Frequencies and percentages will be reported for categorical variables.

## 8.3 Missing data

Missing data will be assessed descriptively by treatment group for each outcome variable in Section 9.1, 9.2, and the potential confounder in Section 7.6.

# 9. STATISTICAL analysis

Statistical analyses will be performed for all primary and secondary outcome variables. Statistical significance will be assessed at the 0.05 level using a two-sided comparative test of treatment effect, comparing the tighter targets group (intervention group) to the less tight targets group (control group), unless otherwise specified. Independent between groups analysis will be the primary method of analysis for all reporting.

Continuous outcomes will be analysed using generalised linear mixed-effects model (GLMM) fitting a normal distribution and identity link function. Marginal ‘least squares’ mean difference and 95% confidence intervals will be estimated from the adjusted model. All model assumptions, including normality, will be assessed.

Binary outcomes will also be analysed using a GLMM fitting a binary distribution and log link function to estimate relative risk with robust 95% confidence intervals. If the model fails to converge a logit link function will be used in the model and odds ratios and 95% confidence intervals presented. Risk differences will be modelled using a binomial distribution with identity link function.

Data points in the control section wedge (Less Tight Target Period) will be compared with data points in the intervention section (Tighter Target Period) to assess the effectiveness of the intervention (37). GLMM will be used to evaluate the main treatment effect.

**Summary of changes between version 1.0 and version 1.1**

1.Secondary outcomes for mothers (page 9), under subheading 11 (Maternal psychological health): SF-36 changed to SF-36 version 2 to clarify which version of the questionnaire was used.

2.Secondary outcomes for children (page 11), under subheading 3 (Neurological status): Q10, 2 = Yes was changed to Q10, 2.1 = Yes to ensure those who had answered Yes to Q10 2.2 or Q10 2.3 and were classified as deaf were not counted in the ‘other hearing problems’ question.

3.Secondary outcomes for children (page 12), under subheading 5 (Behavioural and Emotional Problems): SCQ cut-off changed from ≥11 to ≥15. The sentence “We have chosen a score of ≥15 as this higher cut-off has a higher specificity for a diagnosis of autism spectrum disorder (references)” was added.

# 10. DRAFT SHELL TABLES

## 10.1 Figure 1: Chart of participants in the TARGET 4.5 Year Follow-up Study.

|  | | | **Consented to the TARGET Trial**  **xxx women and xxx infants** | | | | | |  | | | |
| --- | --- | --- | --- | --- | --- | --- | --- | --- | --- | --- | --- | --- |
|  | | | Tight Group  xxx women, xxx infants | | | Less Tight Group  xxx women, xxx infants | | |  | | | |
| xx mother and infant lost to follow-up prior to birth  xx mothers and infants withdrew prior to postnatal discharge | | |  |  | | |  | | |  | xx mothers and infants lost to follow-up prior to birth  Xx mothers and infants withdrew prior to postnatal discharge | |
|  | | | **Considered for eligibility for the TARGET 4.5 Year Follow-up Study**  **xxx women and xxx children** | | | | | |  | | | |
|  | | | Tight Group  Xxx women, xxx children | | | Less Tight Group  Xxx women, xxx children | | |  | | | |
| x mothers and children withdrew after postnatal discharge  x mother and her child in care outside the family  xx mother and child died | |  |  | | |  | | |  | | x mothers and children withdrew after postnatal discharge  xx mother and her child in care outside the family  xx mother and child died | |
|  | | | **Eligible for the TARGET 4.5 Year Follow-up Study**  **xxx women and xxx children** | | | | | |  | | | |
|  | | | Tight Group  Xxx women, xxx children | | | Less Tight Group  Xxx women, xxx children | | |  | | | |
| Xx mothers and children non-contactable  xx mothers and children declined | | |  |  | | |  | | |  | xx mothers and children non-contactable xx mothers and children declined | |
|  | | |  | | |  | | |  | | | |
|  | **Tight Group**  **Xxx women, xxx children** | | | | | **Less Tight Group**  **Xxx women, xxx children** | | | | | |  |
|  | Primary Outcome  Xxx women | | | | Primary Outcome Xxx children | Primary Outcome Xxx women | | Primary Outcome  Xxx children | | | |  |

## 10.2 Table 1: Baseline TARGET Trial entry characteristics of women who were eligible for follow-up at 4.5 years and included or not included.

|  | **Women included** | **Women not included** | **TOTAL** |
| --- | --- | --- | --- |
| Age (years)* |  |  |  |
| Primiparity |  |  |  |
| Gestational age at entry (weeks)^×^ |  |  |  |
| BMI (kg/m^2^)* |  |  |  |
| **BMI category** | | | |
| Underweight (<18.5 kg/m^2^) |  |  |  |
| Normal (18.5-24.9 kg/m^2^) |  |  |  |
| Overweight (25.0-29.9 kg/m^2^) |  |  |  |
| Class I Obesity (30.0-34.9 kg/m^2^) |  |  |  |
| Class II Obesity (35-39.9 kg/m^2^) |  |  |  |
| Class III Obesity (≥40 kg/m^2^) |  |  |  |
| Overweight or Obese (> 25 kg/m^2^) |  |  |  |
| **Ethnicity** | | | |
| Māori |  |  |  |
| Pacifica |  |  |  |
| Asian |  |  |  |
| Other |  |  |  |
| NZ European |  |  |  |
| **NZ Deprivation Category** | | | |
| 1-2 – least deprived |  |  |  |
| 3-4 |  |  |  |
| 5-6 |  |  |  |
| 7-8 |  |  |  |
| 9-10 – most deprived |  |  |  |
| **Diagnostic OGTT^×^** | | | |
| Fasting (mmol/l) |  |  |  |
| 2 hour (mmol/l) |  |  |  |
| **Medical history** | | | |
| Previous GDM |  |  |  |

BMI = body mass index, OGTT = oral glucose tolerance test.

Values are number (%) unless otherwise indicated. * = mean (SD); ^×^ = median (interquartile range).

## 10.3 Table 2: Baseline characteristics of mothers and children included in the 4.5 Year Follow-up.

|  | **Tighter target group** | **Less tight target group** |
| --- | --- | --- |
| **MOTHERS** |  |  |
| Age* |  |  |
| Primiparity |  |  |
| Gestational age at entry to TARGET trial (weeks)^×^ |  |  |
| **BMI category** |  |  |
| BMI (kg/m^2^)* |  |  |
| Underweight (<18.5 kg/m^2^) |  |  |
| Normal (18.5-24.9 kg/m^2^) |  |  |
| Overweight (25.0-29.9 kg/m^2^) |  |  |
| Previous history of GDM prior to index pregnancy |  |  |
| Smoking at trial entry |  |  |
| Class I Obesity (30.0-34.9 kg/m^2^) |  |  |
| Class II Obesity (35-39.9 kg/m^2^) |  |  |
| Class III Obesity (≥40 kg/m^2^) |  |  |
| Overweight/Obese (> 25 kg/m^2^) |  |  |
| **Ethnicity** |  |  |
| Māori |  |  |
| Pacifica |  |  |
| Asian |  |  |
| Other |  |  |
| NZ European |  |  |
| **NZ Deprivation Category** |  |  |
| 1-2 – least deprived |  |  |
| 3-4 |  |  |
| 5-6 |  |  |
| 7-8 |  |  |
| 9-10 – most deprived |  |  |
| **Diagnostic OGTT** |  |  |
| Fasting glucose (mmol/l) |  |  |
| 2 hour glucose (mmol/l) |  |  |
| **GDM treatment during pregnancy** |  |  |
| Metformin |  |  |
| Insulin |  |  |
| Metformin and Insulin |  |  |
| Metformin or Insulin |  |  |
| **Medical history** |  |  |
| Previous history of GDM prior to index pregnancy |  |  |
| Smoker |  |  |
| **CHILDREN** |  |  |
| Gestational age at birth (weeks)^×^ |  |  |
| Male |  |  |
| Birthweight (kg)* |  |  |
| LGA |  |  |
| SGA |  |  |
| Hypoglycaemia |  |  |
| Admission to NICU |  |  |
| Breastfed at hospital discharge |  |  |
| Age at Follow up Assessment |  |  |
| **Ethnicity** |  |  |
| Māori |  |  |
| Pacifica |  |  |
| Asian |  |  |
| Other |  |  |
| NZ European |  |  |

Data are number (%) or mean (SD)*, ^×^ = median (interquartile range). BMI = body mass index, OGTT = oral glucose tolerance test, LGA = large for gestational age, defined as >90^th^ centile using Fenton growth charts (38), SGA = small for gestational age, defined as <10^th^ centile, NICU = Neonatal Intensive Care Unit.

## 10.4 Table 3: Primary and secondary outcomes among the mothers at 4.5 Year Follow-up.

|  | **Total** | **Tighter target group** | **Less tight target group** | **Unadjusted treatment effect**  **(95% CI)** | **P value** | **Adjusted treatment effect (95% CI)** | **P value** |
| --- | --- | --- | --- | --- | --- | --- | --- |
| **Primary Outcomes** | | | | | | | |
| Maternal HbA1c* (mmol/mol) |  |  |  |  |  |  |  |
| **Secondary Outcomes** | | | | | | | |
| **Diabetes or Pre-diabetes** | | | | | | | |
| Any of Pre-diabetes or Diabetes^1^ |  |  |  |  |  |  |  |
| FPG ≥5.6mmol/L^1^ |  |  |  |  |  |  |  |
| Type 1 Diabetes^1^ |  |  |  |  |  |  |  |
| Type 2 Diabetes^1^ |  |  |  |  |  |  |  |
| - Type 2 Diabetes^1^  Treated with lifestyle modification alone |  |  |  |  |  |  |  |
| - Type 2 Diabetes^1^  Treated with metformin or insulin |  |  |  |  |  |  |  |
| - Type 2 Diabetes^1^  Treated with metformin & insulin |  |  |  |  |  |  |  |
| - Type 2 Diabetes^1^  Treated with metformin |  |  |  |  |  |  |  |
| - Type 2 Diabetes^1^  Treated with insulin |  |  |  |  |  |  |  |
| Pre-diabetes^1^ |  |  |  |  |  |  |  |
| **Body size** | | | | | | | |
| Height (m)* |  |  |  |  |  |  |  |
| Weight (kg)* |  |  |  |  |  |  |  |
| BMI (kg/m^2^)* |  |  |  |  |  |  |  |
| -Underweight (<18.5 kg/m^2^) |  |  |  |  |  |  |  |
| -Normal (18.5-24.9 kg/m^2^) |  |  |  |  |  |  |  |
| -Overweight (25.0-29.9 kg/m^2^) |  |  |  |  |  |  |  |
| -Class I Obesity (30.0-34.9 kg/m^2^)^1^ |  |  |  |  |  |  |  |
| -Class II Obesity (35-39.9 kg/m^2^)^1^ |  |  |  |  |  |  |  |
| - Class III Obesity (≥40 kg/m^2^)^1^ |  |  |  |  |  |  |  |
| -Overweight or obese (> 25 kg/m^2^) |  |  |  |  |  |  |  |
| **Plasma lipids*** | | | | | | | |
| Total cholesterol (mmol/L)* |  |  |  |  |  |  |  |
| Triglycerides (mmol/L)* |  |  |  |  |  |  |  |
| HDL result (mmol/L)* |  |  |  |  |  |  |  |
| LDL result (mmol/L)* |  |  |  |  |  |  |  |
| Total cholesterol/HDL ratio |  |  |  |  |  |  |  |
| Triglycerides^1^ >1.7 mmol/L |  |  |  |  |  |  |  |
| HDL^1^ <1.29mmol/L |  |  |  |  |  |  |  |
| Total cholesterol/HDL ratio < 4 mmol/l |  |  |  |  |  |  |  |
| Self-reported dyslipidemia |  |  |  |  |  |  |  |
| On medication |  |  |  |  |  |  |  |
| **Hypertension** | | | | | | | |
| Self-reported hypertension |  |  |  |  |  |  |  |
| On medication |  |  |  |  |  |  |  |
| **Metabolic syndrome**^1^ | | | | | | | |
| Present |  |  |  |  |  |  |  |
| Any further pregnancies |  |  |  |  |  |  |  |
| Number of further pregnancies* |  |  |  |  |  |  |  |
| **-**With GDM |  |  |  |  |  |  |  |
| -With pre-eclampsia |  |  |  |  |  |  |  |
| -With pre-term birth |  |  |  |  |  |  |  |
| **Other** | | | | | | | |
| Current smoker |  |  |  |  |  |  |  |
| **Health related quality of life** | | | | | | | |
| **Self-reported health** | | | | | | | |
| -Excellent |  |  |  |  |  |  |  |
| -Very good |  |  |  |  |  |  |  |
| -Good |  |  |  |  |  |  |  |
| -Fair |  |  |  |  |  |  |  |
| -Poor |  |  |  |  |  |  |  |
| -Declined |  |  |  |  |  |  |  |
| **SF-36** | | | | | | | |
| Overall physical component* |  |  |  |  |  |  |  |
| Overall mental component* |  |  |  |  |  |  |  |
| Physical functioning (10 items)* |  |  |  |  |  |  |  |
| Bodily pain (2 items)* |  |  |  |  |  |  |  |
| Role limitations due to physical health problems (4 items)* |  |  |  |  |  |  |  |
| Role limitations due to personal or emotional problems (3 items)* |  |  |  |  |  |  |  |
| Emotional wellbeing (5 items)* |  |  |  |  |  |  |  |
| Social functioning (2 items)* |  |  |  |  |  |  |  |
| Energy / fatigue (4 items)* |  |  |  |  |  |  |  |
| General health perceptions (5 items)* |  |  |  |  |  |  |  |
| Perceived change in health (1 item)* |  |  |  |  |  |  |  |
| **Emotional wellbeing** | | | | | | | |
| EPDS ≥12 |  |  |  |  |  |  |  |
| EPDS* |  |  |  |  |  |  |  |
| Short form STAI ≥15 |  |  |  |  |  |  |  |
| Short form STAI* |  |  |  |  |  |  |  |
| **Adherence to postnatal screening recommendations^2^** | | | | | | | |
| HbA1c at 3 months and annually |  |  |  |  |  |  |  |
| Proportion of recommended HbA1c measurements completed |  |  |  |  |  |  |  |
| **HbA1c measurement** | | | | | | | |
| -Any |  |  |  |  |  |  |  |
| -One |  |  |  |  |  |  |  |
| -Two |  |  |  |  |  |  |  |
| -Three |  |  |  |  |  |  |  |
| -Four |  |  |  |  |  |  |  |
| -Five |  |  |  |  |  |  |  |
| **Healthcare utilisation** | | | | | | | |
| **Further pregnancies** | | | | | | | |
| Any further pregnancies |  |  |  |  |  |  |  |
| Number of further pregnancies* |  |  |  |  |  |  |  |
| **-**With GDM |  |  |  |  |  |  |  |
| -With pre-eclampsia |  |  |  |  |  |  |  |
| -With pre-term birth |  |  |  |  |  |  |  |
| **Cardiovascular or cerebrovascular events** | | | | | | | |
| Myocardial infarction |  |  |  |  |  |  |  |
| -Age of first myocardial infarction (years) |  |  |  |  |  |  |  |
| -Admitted to hospital with myocardial infarction |  |  |  |  |  |  |  |
| Angina |  |  |  |  |  |  |  |
| Stroke |  |  |  |  |  |  |  |
| -Age of first stroke (years) |  |  |  |  |  |  |  |
| **Any major illnesses** | | | | | | | |
| -Cardiovascular |  |  |  |  |  |  |  |
| -Respiratory |  |  |  |  |  |  |  |
| -Gastroenterology |  |  |  |  |  |  |  |
| -Nephrology |  |  |  |  |  |  |  |
| -Gynaecology |  |  |  |  |  |  |  |
| -Neurology |  |  |  |  |  |  |  |
| -Mental health |  |  |  |  |  |  |  |
| -Musculoskeletal |  |  |  |  |  |  |  |
| -Endocrinology |  |  |  |  |  |  |  |
| -Dermatology |  |  |  |  |  |  |  |
| -Other |  |  |  |  |  |  |  |
| **Diet and Physical Activity** | | | | | | | |
| **Maternal diet: food frequency questionnaire (macronutrients)** | | | | | | | |
| Total Energy (kcals/day)* |  |  |  |  |  |  |  |
| Total Carbohydrate (g/day)* |  |  |  |  |  |  |  |
| Total Fat (g/day)* |  |  |  |  |  |  |  |
| Total Monounsaturated Fat (g/day)* |  |  |  |  |  |  |  |
| Total Polyunsaturated Fat (g/day)* |  |  |  |  |  |  |  |
| Total Protein (g/day)* |  |  |  |  |  |  |  |
| **Maternal physical activity: SQUASH** | | | | | | | |
| Total METs* |  |  |  |  |  |  |  |
| Light intensity (<4 METs) |  |  |  |  |  |  |  |
| Moderate intensity (4-6.5 METs) |  |  |  |  |  |  |  |
| Vigorous intensity (>6.5 METs) |  |  |  |  |  |  |  |

Data are number (%) or mean (SD)*, with treatment effects as relative risks or mean differences and 95% confidence intervals.

^1^Where metabolic syndrome is defined as three or more of: hypertension; triglycerides >1.7mmol/L; HDL-cholesterol <1.29mmol/L; FPG >5.6mmol/L; prediabetes or diabetes, (Laboratory results from maternal information medical records form); obesity (BMI >30kg/m^2^ using height from TARGET Trial and self-reported weight at follow-up) (Alberti 2009, Health Navigator 2023).

^2^ Where adherence to 3 month post-partum HbA1c measurement was defined as having had an HbA1c between 6 weeks to 6 months post-partum, and adherence to annual post-partum HbA1c was having had an HbA1c annually +/- 6 months. Number of HbA1c measurements counted from the birth to 4.5years after the birth or until diagnosed with Type 2 Diabetes

BMI = Body Mass Index, HbA1c = glycated haemoglobin, SF-36 = 36-item short form health related quality of life questionnaire, EPDS = Edinburgh Postnatal Depression Score, STAI = short-form state trait anxiety inventory, MET(s) = the metabolic equivalent of task, a grading of intensity of exercise, SQUASH = activity patterns short questionnaire to assess health enhancing physical activity.

## 10.5 Table 4: Primary and secondary outcomes among the children at 4.5 Year Follow-up.

|  | **Total** | **Tighter target group** | **Less tight target group** | **Unadjusted treatment effect**  **(95% CI)** | **P value** | **Adjusted treatment effect (95% CI)** | **P value** |
| --- | --- | --- | --- | --- | --- | --- | --- |
| **Primary outcome** | | | | | | | |
| Child BMI z -score* |  |  |  |  |  |  |  |
| **Secondary outcomes** | | | | | | | |
| **Body size** | | | | | | | |
| Height (m)* |  |  |  |  |  |  |  |
| Height z-score |  |  |  |  |  |  |  |
| Short Stature (height z-score < -2) |  |  |  |  |  |  |  |
| Weight (kg)* |  |  |  |  |  |  |  |
| Weight z-score |  |  |  |  |  |  |  |
| < -2 |  |  |  |  |  |  |  |
| -2 to +2 |  |  |  |  |  |  |  |
| >+2 to +3 |  |  |  |  |  |  |  |
| > +3 |  |  |  |  |  |  |  |
| BMI (kg/m^2^)* |  |  |  |  |  |  |  |
| Overweight/obese (BMI z-score > +2) |  |  |  |  |  |  |  |
| Overweight (BMI z-score > +2 and < +3) |  |  |  |  |  |  |  |
| Obese (BMI z-score > +3) |  |  |  |  |  |  |  |
| **Neurological status** | | | | | | | |
| Any of cerebral palsy, blindness, deafness or known developmental delay^ |  |  |  |  |  |  |  |
| Any known cerebral palsy ^ |  |  |  |  |  |  |  |
| -Mild cerebral palsy |  |  |  |  |  |  |  |
| -Moderate cerebral palsy |  |  |  |  |  |  |  |
| -Severe cerebral palsy |  |  |  |  |  |  |  |
| Blindness |  |  |  |  |  |  |  |
| Deafness |  |  |  |  |  |  |  |
| Any known cognitive impairment (language, motor or cognitive)^ |  |  |  |  |  |  |  |
| -Language delay |  |  |  |  |  |  |  |
| -Motor delay |  |  |  |  |  |  |  |
| -Cognitive delay |  |  |  |  |  |  |  |
| Co-ordination impairment |  |  |  |  |  |  |  |
| -Difficulty walking |  |  |  |  |  |  |  |
| -Difficulty sitting |  |  |  |  |  |  |  |
| -Difficulty using hands |  |  |  |  |  |  |  |
| -Difficulty with head control |  |  |  |  |  |  |  |
| Visual impairment |  |  |  |  |  |  |  |
| -Can see normally with glasses |  |  |  |  |  |  |  |
| -Unable to see normally with glasses |  |  |  |  |  |  |  |
| -Has a squint in one eye |  |  |  |  |  |  |  |
| -Has a squint in both eyes |  |  |  |  |  |  |  |
| Hearing impairment |  |  |  |  |  |  |  |
| -Difficulty hearing but not using hearing aids |  |  |  |  |  |  |  |
| Attending special care programme |  |  |  |  |  |  |  |
| -Due to visual impairment |  |  |  |  |  |  |  |
| -Due to developmental delay/intellectual delay |  |  |  |  |  |  |  |
| -Due to hearing impairment |  |  |  |  |  |  |  |
| -Due to behavioural disturbance |  |  |  |  |  |  |  |
| -Due to cerebral palsy |  |  |  |  |  |  |  |
| -Other reason |  |  |  |  |  |  |  |
| Supportive care in last 2 years |  |  |  |  |  |  |  |
| -Speech pathology |  |  |  |  |  |  |  |
| -Psychological assessment/intervention |  |  |  |  |  |  |  |
| -Physiotherapy |  |  |  |  |  |  |  |
| -Occupational therapy |  |  |  |  |  |  |  |
| -Other |  |  |  |  |  |  |  |
| **Fine and gross motor function** | | | | | | | |
| Proportion of children with abnormal Little DCDQ score (<67 for boys and <68 for girls) |  |  |  |  |  |  |  |
| Little DCDQ overall score* |  |  |  |  |  |  |  |
| Gross motor subscale score |  |  |  |  |  |  |  |
| **Behavioural and emotional problems** | | | | | | | |
| **SDQ** | | | | | | | |
| Behavioural and emotional problems (score ≥14) |  |  |  |  |  |  |  |
| Total difficulties score* |  |  |  |  |  |  |  |
| Externalising score* |  |  |  |  |  |  |  |
| Internalising score* |  |  |  |  |  |  |  |
| **SCQ** | | | | | | | |
| Likely on the autism spectrum (score ≥11) |  |  |  |  |  |  |  |
| Total score* |  |  |  |  |  |  |  |
| **CEBQ** | | | | | | | |
| Food responsiveness (4 items)* |  |  |  |  |  |  |  |
| Emotional overeating (4 items)* |  |  |  |  |  |  |  |
| Enjoyment of food (4 items)* |  |  |  |  |  |  |  |
| Desire to drink (3 items)* |  |  |  |  |  |  |  |
| Satiety responsiveness (5 items)* |  |  |  |  |  |  |  |
| Slowness in eating (4 items)* |  |  |  |  |  |  |  |
| Emotional undereating (4 items)* |  |  |  |  |  |  |  |
| Food fussiness (7 items)* |  |  |  |  |  |  |  |
| **Functional health and wellbeing (CHQ)** | | | | | | | |
| Low physical functioning (score <40 |  |  |  |  |  |  |  |
| Low psychosocial functioning (score <40) |  |  |  |  |  |  |  |
| Physical functioning summary scale score* |  |  |  |  |  |  |  |
| Psychosocial functioning summary scale score* |  |  |  |  |  |  |  |

Data are number (%) or mean (SD)*, with treatment effects as relative risks or mean differences and 95% confidence intervals. × = median (interquartile range). ^ Composite of adverse neurological outcome

For body size we have used WHO charts (3).

Little DCDQ = Little Developmental Coordination Disorder Questionnaire, SDQ = Strength and Difficulties Questionnaire, SCQ = Social Communication Questionnaire, CEBQ = Child Eating Behaviour Questionnaire, CHQ-PF28 = Child Health Questionnaire,

# 11. REFERENCES

1. Crowther CA, Alsweiler JM, Hughes R, Brown J, for the Target Study G. Tight or less tight glycaemic targets for women with gestational diabetes mellitus for reducing maternal and perinatal morbidity? (TARGET): study protocol for a stepped wedge randomised trial. BioMed Central Pregnancy and Childbirth. 2018;18(1):425.

2. Crowther CA, Samuel D, Hughes R, Tran T, Brown J, Alsweiler JM. Tighter or less tight glycaemic targets for women with gestational diabetes mellitus for reducing maternal and perinatal morbidity: A stepped-wedge, cluster-randomised trial. PLoS medicine. 2022;19(9):e1004087.

3. World Health Organization. Body mass inder-for-age (BMI-for-age). Tables: z-scores. Geneva: World Health Organization; 2006.

4. The Ministry of Health. The B4 school check: a handbook for practitioners [Internet]. Wellington: New Zealand Government; 2008 [cited 23 April 2023]. Available from: <https://www.moh.govt.nz/notebook/nbbooks.nsf/0/9de5d356a2c8f2cacc2577140005fad9/$FILE/b4sc-practitionershandbook-march2010.pdf>

5. Freedman DS, Lawman HG, Galuska DA, Goodman AB, Berenson GS. Tracking and variability in childhood levels of BMI: The Bogalusa Heart Study. Obesity (Silver Spring, Md). 2018;26(7):1197-202.

6. New Zealand Guidelines Group. Guidance on the management of type 2 diabetes [Internet]. Wellington: New Zealand Guidelines Group.; 2011 [cited 24 April 2023]. Available from: <https://www.moh.govt.nz/notebook/nbbooks.nsf/0/60306295DECB0BC6CC257A4F000FC0CB/$file/NZGG-management-of-type-2-diabetes-web.pdf>

7. Alberti KG, Eckel RH, Grundy SM, Zimmet PZ, Cleeman JI, Donato KA, et al. Harmonizing the metabolic syndrome: a joint interim statement of the International Diabetes Federation Task Force on Epidemiology and Prevention; National Heart, Lung, and Blood Institute; American Heart Association; World Heart Federation; International Atherosclerosis Society; and International Association for the Study of Obesity. Circulation. 2009;120(16):1640-5.

8. Metabolic syndrome 2022 [02 August 2022]. Available from: <https://healthnavigator.org.nz>

9. Sam CH, Skeaff S, Skidmore PM. A comprehensive FFQ developed for use in New Zealand adults: reliability and validity for nutrient intakes. Public Health Nutrition. 2014;17(2):287-96.

10. Wendel-Vos GC, Schuit AJ, Saris WH, Kromhout D. Reproducibility and relative validity of the short questionnaire to assess health-enhancing physical activity. Journal of Clinical Epidemiology. 2003;56(12):1163-9.

11. Ware JE, Jr., Sherbourne CD. The MOS 36-item short-form health survey (SF-36). I. Conceptual framework and item selection. Medical Care. 1992;30(6):473-83.

12. Cox JL, Holden JM, Sagovsky R. Detection of postnatal depression. Development of the 10-item Edinburgh Postnatal Depression Scale. The British Journal of Psychiatry. 1987;150:782-6.

13. Marteau TM, Bekker H. The development of a six-item short-form of the state scale of the Spielberger State-Trait Anxiety Inventory (STAI). Br J Clin Psychol. 1992;31(3):301-6.

14. Crowther CA, Hiller JE, Moss JR, McPhee AJ, Jeffries WS, Robinson JS. Effect of treatment of gestational diabetes mellitus on pregnancy outcomes. New England Journal of Medicine. 2005;352(24):2477-86.

15. New Zealand Society for the Study of Diabetes. Type 2 diabetes management guidelines. Screening for diabetes in asymptomatic adults: Ministry of Health; [cited 2023 8 May].

16. World Health Organization. Training course on child growth assessment: Introduction. Geneva: World Health Organization; 2008.

17. Wilson BN, Creighton D, Crawford SG, Heath JA, Semple L, Tan B, et al. Psychometric Properties of the Canadian Little Developmental Coordination Disorder Questionnaire for Preschool Children. Physical & Occupational Therapy in Pediatrics. 2015;35(2):116-31.

18. Hudson KN, Willoughby MT. Evaluating the factor structure and criterion validity of the Canadian Little DCDQ: associations between motor competence, executive functions, early numeracy skills, and ADHD in early childhood. Assessment. 2022;29(6):1134-43.

19. Goodman R. The Strengths and Difficulties Questionnaire: a research note. Journal of Child Psychology and Psychiatry, and Allied Disciplines. 1997;38(5):581-6.

20. Rutter M, Bailey, A. and Lord, C. The social communication questionnaire: manual. Los Angeles, CA.: Western Psychological Services.; 2003.

21. Wardle J, Guthrie CA, Sanderson S, Rapoport L. Development of the Children's Eating Behaviour Questionnaire. Journal of Child Psychology and Psychiatry, and Allied Disciplines. 2001;42(7):963-70.

22. Goodman R. Scoring the strengths and difficulties questionnaire for age 4-17 or 18+: Youthinmind; 2016 [cited 2023]. Available from: <https://socwel.ku.edu/sites/socwel/files/documents/Research%20Projects/Family%20First/Survey%20Measures/SDQ_English(USA)_4-17scoring_repaired%20-%20Remediated.pdf>

23. Wiggins LD, Bakeman R, Adamson LB, Robins DL. The utility of the social communication questionnaire in screening for autism in children referred for early intervention. Focus on Autism and Other Developmental Disabilities. 2007;22(1):33-8.

24. Moody EJ, Reyes N, Ledbetter C, Wiggins L, DiGuiseppi C, Alexander A, et al. Screening for Autism with the SRS and SCQ: Variations across Demographic, Developmental and Behavioral Factors in Preschool Children. J Autism Dev Disord. 2017;47(11):3550-61.

25. Berument SK, Rutter M, Lord C, Pickles A, Bailey A. Autism screening questionnaire: Diagnostic validity. British Journal of Psychiatry. 1999;175(5):444-51.

26. PhenX Toolkit. Protocol - Child Eating Behaviour Questionnaire (CEBQ) 2023 [April 24 2023]. Available from: <https://www.phenxtoolkit.org/protocols/view/650301>

27. Landgraf JM, Abetz L, Ware JE. Child health questionnaire (CHQ) : a user's manual. Boston, Mass.: Landgraf & Ware; 1999.

28. Hussey MA, Hughes JP. Design and analysis of stepped wedge cluster randomized trials. Contemporary Clinical Trials. 2007;28(2):182-91.

29. Mdege ND, Man MS, Taylor Nee Brown CA, Torgerson DJ. Systematic review of stepped wedge cluster randomized trials shows that design is particularly used to evaluate interventions during routine implementation. Journal of Clinical Epidemiology. 2011;64(9):936-48.

30. Woertman W, de Hoop E, Moerbeek M, Zuidema SU, Gerritsen DL, Teerenstra S. Stepped wedge designs could reduce the required sample size in cluster randomized trials. Journal of Clinical Epidemiology. 2013;66(7):752-8.

31. Hussey M, Hughes J. Design and analysis of stepped wedge cluster randomized trials. Contemp Clin Trials. 2007;28(2):182-91. Epub 2006 Jul 7.

32. Woertman W. Stepped wedge designs could reduce the required sample size in cluster randomized trials. J Clini Epidemiol. 2013;66(7):752-8.

33. Mdege ND, Man M-S, Taylor CA, Torgerson DJ. Systematic review of stepped wedge cluster randomized trials shows that design is particularly used to evaluate interventions during routine implementation. Journal of Clinical Epidemiology. 2011;64(9):936-48.

34. McKinlay CJD, Alsweiler JM, Anstice NS, Burakevych N, Chakraborty A, Chase JG, et al. Association of neonatal glycemia with neurodevelopmental outcomes at 4.5 years. JAMA Pediatrics. 2017;171(10):972-83.

35. NCSS Statistical Software. PASS (Power Analysis and Sample Size) Kaysville, Utah, USA: NCSS Statistical Software; [27 April 2023]. Available from: ncss.com/software/pass

36. Lumley T, Diehr P, Emerson S, Chen L. The importance of the normality assumption in large public health data sets. Annual Review of Public Health. 2002;23:151-69.

37. Brown CA, Lilford RJ. The stepped wedge trial design: a systematic review. BMC medical research methodology. 2006;6:54.

38. Fenton TR, Kim JH. A systematic review and meta-analysis to revise the Fenton growth chart for preterm infants. BMC pediatrics. 2013;13:59.
